# A role for human homologous recombination factors in suppressing microhomology-mediated end joining

**DOI:** 10.1093/nar/gkw326

**Published:** 2016-04-29

**Authors:** Sara Ahrabi, Sovan Sarkar, Sophia X. Pfister, Giacomo Pirovano, Geoff S. Higgins, Andrew C.G. Porter, Timothy C. Humphrey

**Affiliations:** 1 CRUK MRC Oxford Institute for Radiation Oncology, Department of Oncology, University of Oxford, Oxford, OX3 7DQ, UK; 2 Gene Targeting Group, Centre for Haematology, Imperial College Faculty of Medicine, London W12 0NN, UK

## Abstract

DNA double-strand breaks (DSBs) are toxic lesions, which if improperly repaired can result in cell death or genomic instability. DSB repair is usually facilitated by the classical non-homologous end joining (C-NHEJ), or homologous recombination (HR) pathways. However, a mutagenic alternative NHEJ pathway, microhomology-mediated end joining (MMEJ), can also be deployed. While MMEJ is suppressed by C-NHEJ, the relationship between HR and MMEJ is less clear. Here, we describe a role for HR genes in suppressing MMEJ in human cells. By monitoring DSB mis-repair using a sensitive HPRT assay, we found that depletion of HR proteins, including BRCA2, BRCA1 or RPA, resulted in a distinct mutational signature associated with significant increases in break-induced mutation frequencies, deletion lengths and the annealing of short regions of microhomology (2–6 bp) across the break-site. This signature was dependent on CtIP, MRE11, POLQ and PARP, and thus indicative of MMEJ. In contrast to CtIP or MRE11, depletion of BRCA1 resulted in increased partial resection and MMEJ, thus revealing a functional distinction between these early acting HR factors. Together these findings indicate that HR factors suppress mutagenic MMEJ following DSB resection.

## INTRODUCTION

DNA double strand breaks (DSBs) are deleterious lesions that if left unrepaired can lead to cell death, while if mis-repaired can give rise to genomic instability, thus leading to tumorigenesis (1). To survive such lesions and preserve genome integrity, cells possess two main evolutionarily conserved DSB repair mechanisms, namely homologous recombination (HR), and non-homologous end joining (NHEJ) (2). Other repair pathways generally referred to as alternative non-homologous end joining pathways (Alt-NHEJ) (3–5), have been of recent interest. A subset of these repair mechanisms relies on regions of microhomology on either side of the break, which anneal following limited resection in a process called microhomology-mediated end joining (MMEJ) (6–8).

HR is an error-free DSB repair pathway that proceeds through three phases. In mammalian cells the presynaptic phase is triggered by a two-step 5′ to 3′ end resection that produces 3′ single-stranded DNA (ssDNA) overhangs. Resection is initiated by the endonucleolytic activity of the MRE11-RAD50-NBS1 (MRN) complex and the C-terminal binding protein interacting protein (CtIP), which exposes short ssDNA tails (9,10). These become substrates for the extensive resection mediators, Exo1, DNA2 and BLM (11,12). BRCA1 also facilitates the initial resection step of HR (13,14) in conjunction with MRN (15) and CtIP (15,16), where it accelerates the DSB resection rate (17). The exposed ssDNA is initially protected by Replication Protein A (RPA) (18), which is then displaced by RAD51, following its recruitment by BRCA2, to form a nucleoprotein filament (19). The RAD51 nucleofilament promotes strand invasion of the undamaged sister chromatid, which is used as a repair template, resulting in a displacement loop (D-loop). During the synaptic phase of HR, the 3′ end is extended by DNA replication, which can subsequently proceed through a number of sub-pathways. During DSB repair, second end capture and annealing results in double Holliday junction (HJ) formation. In the post-synaptic phase of HR, HJ structures can be resolved with or without crossovers, or dissolved, thus preventing crossovers (20,21). Alternatively, during synthesis-dependent strand annealing (SDSA) (22), the invading and extended strand is expelled from the D-loop to anneal to the second end which, following gap filling and ligation, results in error-free repair (23).

Classical NHEJ (C-NHEJ) is triggered by recognition and protection of DNA ends by the Ku70/Ku80 heterodimer, which forms a ring that encircles duplex DNA. This protects ends from resection and creates a platform to recruit the DNA-PK catalytic subunit (DNA-PKcs) (24,25). Broken ends are then trimmed by Artemis and ligated by DNA Ligase 4 (Lig 4), X-ray repair cross-complementing protein 4 (XRCC4) complex, and XRCC4-like factor (XLF), depending on the nature of the damage (25–27). Although end-protection by Ku in this pathway minimizes resection, thus promoting error-free end joining, this pathway is widely referred to as error-prone as it ligates the ends in a homology-independent fashion potentially leading to small insertions, and/or deletions (indels) at the DSB sites. From a genome-wide perspective, however, C-NHEJ is not as threatening as alternative NHEJ (Alt-NHEJ) pathways for mammalian genome stability (24) and is even considered as a guardian of genome stability (28).

Alt-NHEJ refers to DSB end joining pathways that are independent of the C-NHEJ factors Ku70/Ku80, DNA-PKcs and DNA Lig4. Unlike C-NHEJ, these pathways are highly mutagenic, always associated with indels and commonly lead to chromosomal rearrangements. Importantly, a sub pathway of Alt-NHEJ events termed microhomology mediated end joining (MMEJ) rejoins the ends by base pairing between microhomologous sequences. MMEJ is mediated via CtIP, MRN complex, Poly [ADP-ribose] polymerases1 (PARP-1) and DNA ligase 3 (Lig 3) (29,30). In mammalian cells, PARP-1, a key component of the MMEJ pathway (31), initially competes with Ku heterodimer for binding to DNA ends resulting in PAR formation (32,33). This in turn promotes MRE11 recruitment and the initiation of resection by MRN complex and CtIP (34). Subsequently, DNA end ligation is mediated through DNA Lig 3 (7,35). Further, recent studies have identified a low fidelity DNA polymerase θ (Polθ also known as POLQ) (36) as an important MMEJ factor in mammalian cells. POLQ is recruited to DSB ends by PARP-1 where it facilitates end joining and microhomology annealing (37). While absent from yeast, the role for POLQ in MMEJ appears to be conserved in flies, worms, mice and humans (38). MMEJ generates deletions and translocations at the break points and thus is highly mutagenic (39). However, there are other types of Alt-NHEJ events that do not rely on such microhomologies. The ligation step in these pathways is facilitated through DNA ligase 1(Lig1) (6).

Impairment of a DSB repair pathway is a common feature in human cancers, rendering their survival highly dependent on secondary DSB repair pathways (40). Therefore, studies of the relationships between different DSB repair machineries are of interest as they can provide insight into how to target these defects for cancer therapy purposes. In this regard, HR has an inhibitory impact on C-NHEJ via initiation of resection (41). Conversely, C-NHEJ suppresses HR and Alt-NHEJ by Ku binding and preventing the resection initiation (42,43). However, the exact nature of the interplay between HR and Alt-NHEJ in human cells still remains elusive. A recent study in MEFs (Mouse Embryonic Fibroblast Cells) found elevated levels of PARP-1-dependent translocations following loss of the HR factor RAD54 (44). Moreover, a study in *Saccharomyces cerevisiae (S. cerevisiae)* showed that RPA impairs MMEJ by removing the secondary structures of ssDNA, which facilitates RAD51 filament assembly leading to HR and thereby suppression of annealing of the terminal microhomologies (45). On the other hand, two recent studies in mammalian cells suggested that POLQ-mediated Alt-NHEJ suppresses HR repair mechanisms (46,47). A role for BRCA1 in promoting NHEJ fidelity has been proposed (48). However, studies in MEFs have also shown that BRCA1 promotes Alt-NHEJ at uncapped telomeres (49).

Here, we have investigated the relationship between HR and MMEJ repair of DSBs. We have used a highly sensitive HPRT-based assay (50,51) together with other GFP-based reporter assays (52–54) to characterize DSB mis-repair events observed following depletion or inhibition of HR and NHEJ factors. Following both physical and genetic analyses, we have established a role for HR factors in suppressing MMEJ at break point junctions. Further, our data suggest the existence of POLQ-dependent and POLQ-independent MMEJ pathways, both of which are suppressed by RPA. Last, we define a role for BRCA1 in functioning downstream of CtIP and MRE11 to promote resection, thereby preserving genome integrity by counteracting MMEJ.

## MATERIALS AND METHODS

### Cell culture

HT1080 (human fibrosarcoma, HPRT assay), U2OS (human osteosarcoma, GFP-based HR reporter (52)), and H1299 (human non-small cell lung carcinoma, GFP-based C-NHEJ reporter (54)) cells were cultured as described previously (51,55). C-NHEJ reporter cells were a kind gift from Atsushi Shibata and Takashi Kohno. MMEJ reporter cells (U2OS with integrated EJ2-GFP reporter) were a kind gift from Jeremy Stark (53) and were grown in high glucose DMEM supplemented with L-glutamine, 10% fetal bovine serum, and 1% Pen/Strep solution (10 000 U/ml penicillin, 10 000 mg/ml streptomycin), including 8 mg/ml plasmocin and 2 mg/ml puromycin.

### HAT to select for HPRT gain of function

HAT selection was carried out by adding 1× HAT supplement (Invitrogen) directly to the DMEM medium for 10 days before the I-SceI transfection.

### 6-TG to select for HPRT loss of function

6-TG selective medium was made by making up 1000 × 6-TG of 15 mg/ml by dissolving the 6-TG powder (sigma) in 1N NaOH and ddH2O. 1000 × 6-TG was added to DMEM and used at a final concentration of 15 mg/ml. 6-TG medium was added 5 days after the I-SceI transfections.

### HPRT-based I-SceI-cleavable reporter assay

The assay was based on human fibrosarcoma (HT1080) cells with a functional but I-SceI-cleavable *HPRT* gene (clone 5.2.1), as described previously (50). Use of this assay in conjunction with siRNA knockdowns has been previously described (51). Briefly, an I-SceI cut site was targeted into exon 6 of the endogenous human*HPRT* gene, without disrupting the functionality of the gene. These cells were seeded in 6-well plates and were immediately transfected with siRNAs using RNAiMAX from Invitrogen according to the manufacturer's instructions. The following day, medium was replaced with fresh DMEM. 48 h after siRNA transfections, cells were lipofected with I-SceI plasmid, which was performed in a ratio of 6 μg DNA: 18 μl Fugene in a total of 300 μl transfection mix in DMEM with no Pen/Strep and fetal bovine serum to transfect each well of a 6-well plate using the Fugene 6 transfection reagent (Promega). 24 h later, medium was replaced with fresh DMEM. Five days after the I-SceI transfection, the cells were seeded in different densities within 100 mm plates (i.e., 10^5^, 5 × 10^4^, 10^4^, and 10^3^ per plate). Cells used to determine the mutation frequencies were then exposed to 6-TG selection, while the cells used for determining plating efficiencies were kept in non-selective media. 6-TG medium was refreshed every 2–3 days. Cells were incubated for 10–12 days to form colonies, which were stained and counted for the purpose for calculating the mutation frequencies. Further, a total of 30 clones from each genetic background were isolated and grown in 48-well plates. These were subsequently expanded into 24-well plates. At full confluency cells for each clone were then trypsinised and collected for extraction of genomic DNA using a Qiagen Flexi gene DNA kit according to the manufacturer's instructions. Genomic DNA was then quantified and used for PCR amplification across the I-SceI break site and sequencing. Primers and PCR conditions to amplify across the I-SceI site have been previously described (51).

### Data analysis

Sequence alignment was conducted using DNA Data Bank of Japan (DDBJ) ClustalW program (version 2.1).

### Graphical display of results and statistical analysis

For all statistical analysis and graphical display, the program GraphPad Prism (www.graphpad.com) was used.

### siRNA transfections

HT1080 or U2OS or H1299 cells were transfected with siRNAs (10 nM final concentration) using RNAiMAX (Invitrogen) according to the manufacturer's instructions. Medium was replaced 24 h after transfection. The sequences of the siRNAs are listed below:

non-targeting (NT) proprietary sequence of supplier (Qiagen),

BRCA2 (Thermo Scientific): GGGAAACACUCAGAUUAAAUU, UUUAAUCUGAGUGUUUCCCUU;

BRCA1 (Dharmacon): AAUGCCAAAGUAGCUAAUGUAUUUU, AAUACAUUAGCUACUUUGGCAUUUU;

CtIP (Dharmacon): GAGGUUAUAUUAAGGAAGA, GGAGCUACCUCUAGUAUCA, GAACAGAAUAGGACUGAGU, GCACGUUGCCCAAAGAUUC;

MRE11 (Dharmacon): GGAGGUACGUCGUUUCAGA, GGAAAUGAUACGUUUGUAA, CGAAAUGUCACUACUAAGA, GAAAGGCUCUAUCGAAUGU;

RPA (Dharmacon): AACUGGUUGACGAAAGUGGUGUU, CACCACUUUCGUCAACCAGUUUU;

POLQ (Ambion, Lifetechnologies): CCGCUUUUGGAGUCAGUAATT, UUACUGACUCCAAAAGCGGTA.

### Western blotting

Whole cell extracts and the protein concentration measurements for western blotting were performed as described previously (51). Equal amounts of protein were separated on NuPAGE Tris-Acetate or Bis-Tris gel (Invitrogen) and transferred to a PVDF membrane (0.45 μm pore size) (Invitrogen). After blocking, the membranes were incubated with appropriate primary antibodies at 4°C overnight followed by secondary antibodies at room temperature for 1 h. Imaging of protein was performed by the BIO-RAD ChemiDoc Imaging sytem.

Primary antibodies used for western blotting are listed below:

BRCA2 (Santa Cruz, sc-28235); BRCA1 (Calbiochem, OP92); RAD51 (Santa Cruz, sc-8349); CtIP (GeneTex 19E8); MRE11 (Abcam, ab33125); RPA32 (Abcam, 2175); Tubulin (Sigma, T5168). All secondary antibodies were purchased from Invitrogen.

### Inhibitors

The PARP-1 inhibitor, Olaparib (AZD 2281) was dissolved in DMSO and used at 5 μM final concentration. The DNA-PKcs inhibitor, NU7441 (Axon) was dissolved in DMSO and used at 5, and 15 μM final concentrations.

### Quantitative RT-PCR

RNA was extracted using an RNeasy mini kit (Qiagen). A SuperScript® VILOTM (Invitrogen/Life Technologies) cDNA synthesis kit was used to reverse transcribe cDNA from total RNA according to manufacturer's instructions. Quantitative PCR was performed using 7500 Fast Real-Time PCR detection system (Applied Biosystems). Reactions (25 μl each) were prepared in triplicate in a 96-well reaction plate. Each reaction contained 20 ng cDNA, 200 nM of each primer, 10 μl water and 12.5 μl Absolute Blue QPCR SYBR low ROX Mix (Thermo Scientific). DNA levels were normalized to the GAPDH calculated using a 2-ΔΔCt method. QPCR settings were as follows: Initialization at 95°C for 15 min, denaturation at 95°C for 15 s, annealing at 60°C for 30 s, and extension at 72°C for 30 s and repeat for 40 cycles. Primers used for the qRT-PCR are listed below:

qRT-PCR F: 5′-AGT CGC ACA CTG CTA CAG GAC GA-3′

qRT-PCR R: 5′-GGC ACA AAG GCA TGT CGC ATG C-3′

### GFP-based I-SceI-cleavable reporter assays (HR, C-NHEJ and MMEJ)

To examine the effects of gene loss of function on the HR, C-NHEJ, and MMEJ repair pathways a panel of GFP-based I-SceI-cleavable reporter cell lines was used. The cells were transfected with an I-SceI plasmid using Lipofectamine 2000 (Invitrogen) according to the manufacturer's instructions in 6-well plates 48 h after the siRNA knockdowns. After 2 days, the cells from each well were trypsinised and dispersed in 200 µl medium. The 400 µl suspension was then immediately mixed with 200 µl of 10% formaldehyde, and vortexed for 2–3 s before FACS (FACSCalibur) to quantitate the number of GFP positive cells. For FACS analysis, non-overlapping gates were defined by three populations of cells from the same cell line: normal cells, cells transfected with EGFP or DsRed plasmids. 20 000 cells were analyzed for each sample.

## RESULTS

### BRCA2 depletion promotes DSB-induced deletions typical of MMEJ

We have previously shown that siRNA-mediated depletions of SETD2 or RAD51 had similar effects on I-SceI-induced DSBs in the *HPRT* gene, namely an increased overall frequency of *HPRT* mutagenesis associated with increases in both deletion lengths and use of microhomologies on either side of the deletions (51). These findings suggested a possible role for HR in suppressing MMEJ.

**Figure 1.**
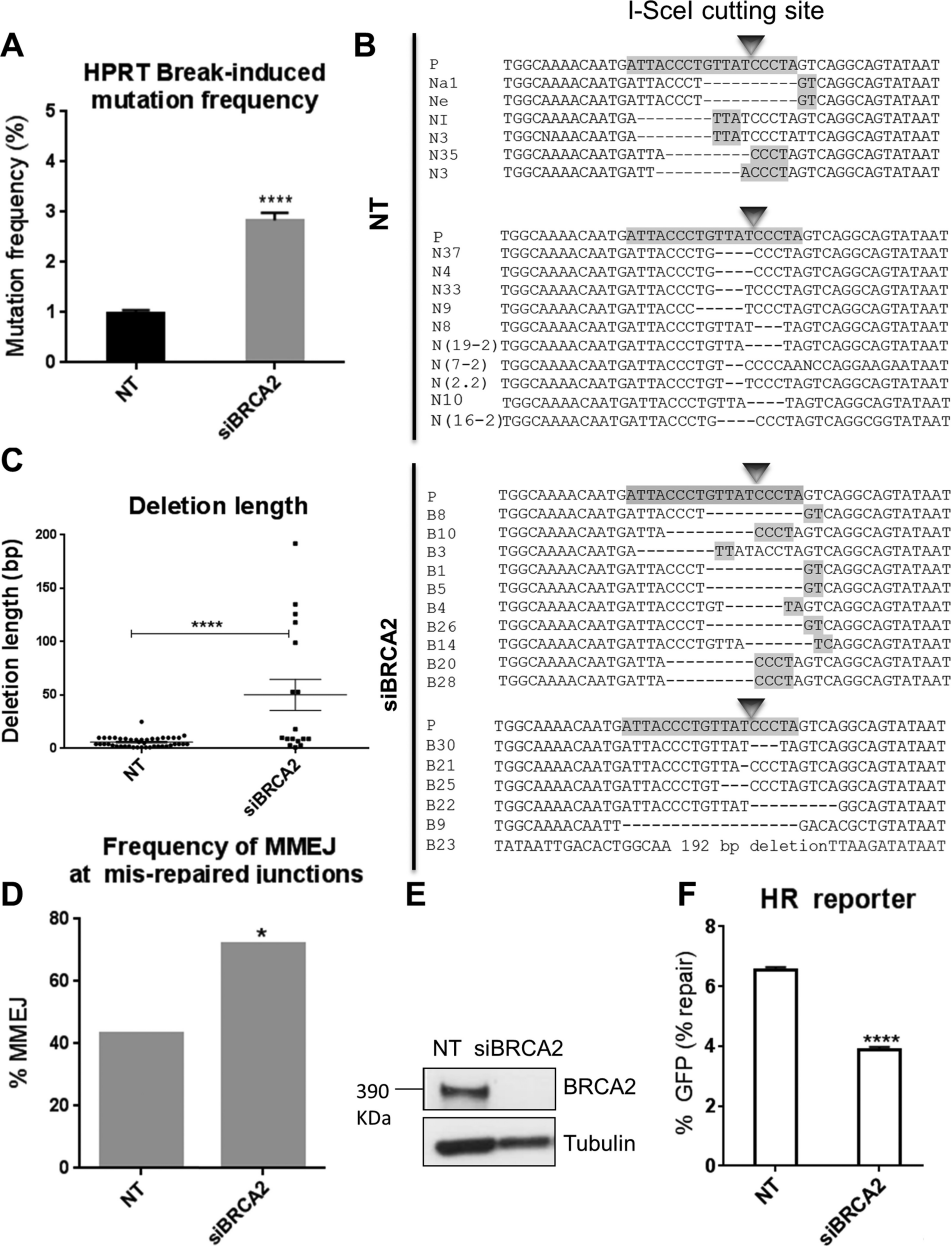
BRCA2 depletion promotes DSB-induced deletions typical of MMEJ. (**A**) Break-induced mutation frequency of HPRT reporter cells treated with NT control siRNA (NT), and BRCA2 siRNA (siBRCA2). Error bars represent SEM from three independent experiments. ****P < 0.0001. (**B**) Representative sequence alignments of the PCR products in NT control (NT), and BRCA2-depleted (siBRCA2) cells isolated from three independent experiments (see also Supplementary Figures S1A, S2A–C and S3). I-SceI recognition sequence and terminal microhomologies at the break sites are highlighted. (**C**) Average length of deletions (bp) in different genetic backgrounds. Each dot represents an independent clone. The lines represent mean and SEM, **** *P* < 0.0001. (**D**) Frequency of MMEJ at mis-repaired junctions in HPRT deletion mutants isolated from cells treated with NT control siRNA (NT) or BRCA2 siRNA (siBRCA2). *P* values calculated by statistical analysis ‘‘difference between proportions’’, * *P* < 0.05. (E) Western blot showing BRCA2 knockdown in HT1080 cells 48 h following siRNA transfection. (F) HR repair efficacy of DR-GFP reporter cells treated with NT control siRNA (NT), and BRCA2 siRNA (siBRCA2), indicated by the percentage of GFP-positive cells. Error bars show SEM from three independent experiments, **** *P* < 0.0001.

To test this further, we applied the same approach (Supplementary Figure S1) to assess a possible role for other HR factors in suppressing MMEJ. BRCA2, a key recombination mediator in mammalian cells, facilitates RAD51 loading onto ssDNA. We therefore anticipated that BRCA2 might also suppress MMEJ. We first depleted BRCA2 and measured the frequency of *HPRT* inactivation (mutation frequency) following I-SceI-induced DSBs. Knockdown of BRCA2 resulted in a significantly increased I-SceI-induced mutation frequency (2.8%; *P* < 0.0001), compared to cells treated with non-targeting control (NT) siRNA (1.1%) (Figure 1A). Sequence analysis of HPRT-negative clones derived from cells treated with NT siRNA (Figure 1B, and Supplementary Figure S2A–C) or BRCA2 siRNA (Figure 1B, and Supplementary Figure S3) indicated average deletion lengths of 5.6 and 49 bp, respectively. Thus BRCA2 depletion resulted in a 9-fold increase in deletion lengths (*P* < 0.0001) (Figure 1C). Insertions at the HPRT break site were also detected but their frequencies were not significantly altered by BRCA2-depletion (Supplementary Figure S14).

Further examination of the sequences at deletion junctions showed that the proportion of deletions appearing to have arisen by MMEJ rose significantly from 43%, for cells treated with NT control siRNA, to 72% for cells treated with BRCA2 siRNA (*P*< 0.05) (Figure 1D). BRCA2 depletion in HT1080 cells was confirmed by western blot (Figure 1E). To assess the effect of BRCA2 depletion on HR, we used a previously described (51) U2OS cell line carrying a well-characterized GFP-based reporter for HR (DR-GFP) (52). We found that the frequency of HR in BRCA2-depleted cells was significantly reduced by 41% compared to the NT controls (*P*< 0.0001), consistent with a role for BRCA2 in promoting HR (19) (Figure 1F). Thus BRCA2 depletion causes both a decrease in HR and an apparent increase in MMEJ suggesting roles for BRCA2 not only in promoting HR but also in suppressing MMEJ.

### BRCA1 depletion promotes DSB-induced deletions typical of MMEJ

**Figure 2.**
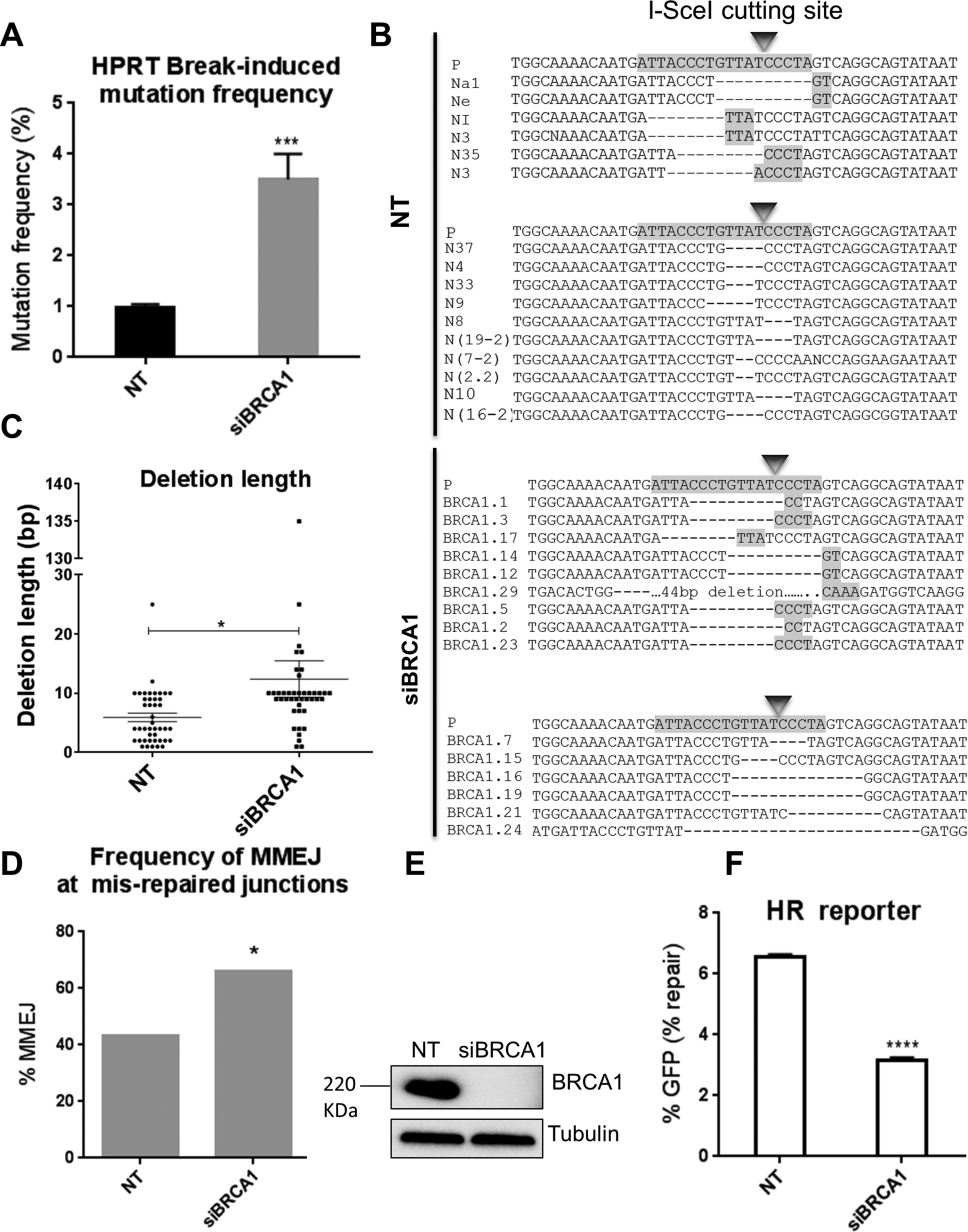
BRCA1 depletion promotes DSB-induced deletions typical of MMEJ. (**A**) Break-induced mutation frequency of HPRT reporter cells treated with NT control siRNA (NT), BRCA1 siRNA (siBRCA1). Error bars represent SEM from three independent experiments. *** *P* < 0.001. (**B**) Representative sequence alignments of the PCR products in NT control (NT), and BRCA1-depleted (siBRCA1) cells from three independent experiments (see also Supplementary Figures S1A, and S4). I-SceI recognition sequence and terminal microhomologies at the break sites are highlighted. (**C**) Average length of deletions (bp) in different genetic backgrounds. Each dot represents an independent clone. The lines represent mean and SEM, *P < 0.05, ***P < 0.001. (**D**) Frequency of MMEJ at mis-repaired junctions in HPRT deletion mutants isolated from cells treated with NT control siRNA (NT) or BRCA1 siRNA (siBRCA1). *P* values calculated by statistical analysis ‘‘difference between proportions’’, *P < 0.05. (**E**) Western blot showing BRCA1 knockdown in HT1080 cells 48 h following siRNA transfection. (**F**) HR repair efficacy of DR-GFP reporter cells treated with NT control siRNA (NT), and BRCA1 siRNA (siBRCA1), indicated by the percentage of GFP-positive cells. Error bars show SEM from three independent experiments, **** *P* < 0.0001.

BRCA1 is also an essential factor for HR but has a distinct function in HR compared to BRCA2, where BRCA1 facilitates 5′ to 3′ resection of DSBs to generate 3′ ssDNA tails (56). To investigate a possible role for BRCA1 in suppressing MMEJ, we examined the effect of BRCA1 depletion on DSB mutational signatures in the HPRT reporter system. Like BRCA2 depletion, BRCA1 depletion resulted in a significantly enhanced mutation frequency (3.5%; *P* = 0.001) following I-SceI-induced DSBs compared to that of NT controls (1.1%) (Figure 2A). Further, sequencing of individually isolated HPRT mutated clones from a BRCA1-depleted background (Figure 2B and Supplementary Figure S4) indicated an average deletion length of 17 bp, significantly (3-fold) greater than the deletion lengths in NT control siRNA-treated cells (*P* = 0.04) (Figure 2C). Interestingly, the deletion lengths in BRCA1-depleted cells were significantly (2.8-fold) smaller than to those in BRCA2-depleted cells (*P* < 0.05). This is consistent with the early role for BRCA1 in promoting resection during HR, prior to BRCA2-assisted RAD51 loading. Insertions at the HPRT break site were also detected but their frequency was not significantly altered by BRCA1-depletion (Supplementary Figure S14). Among the sequenced junctions in HPRT negative cells, 66% (*P*< 0.05) appeared to have been generated by MMEJ following BRCA1 depletion, a 1.5-fold increase over the 43% in cells with normal BRCA1 levels (Figure 2D). Therefore, BRCA1 depletion, like BRCA2-depetion, resulted in a significant increase in the proportion of mutagenic NHEJ events appearing to occur by MMEJ (Figure 2D). BRCA1 depletion in HT1080 cells was confirmed by western blot (Figure 2E). Consistent with the role of BRCA1 in HR (14), HR was significantly reduced by 52% (*P* < 0.0001) in the DR-GFP reporter (U2OS cells) (52) following BRCA1 knockdown (Figure 2F). Thus, BRCA1 depletion results in reduced HR repair and significantly increased mutation frequency associated with a larger proportion of MMEJ events. Together with the similar effects of BRCA2, RAD51 and SETD2 depletion, these results suggest a common role for HR factors in suppressing MMEJ.

### MMEJ-like DSB repair in HR deficient cells is independent of DNA-PKcs

**Figure 3.**
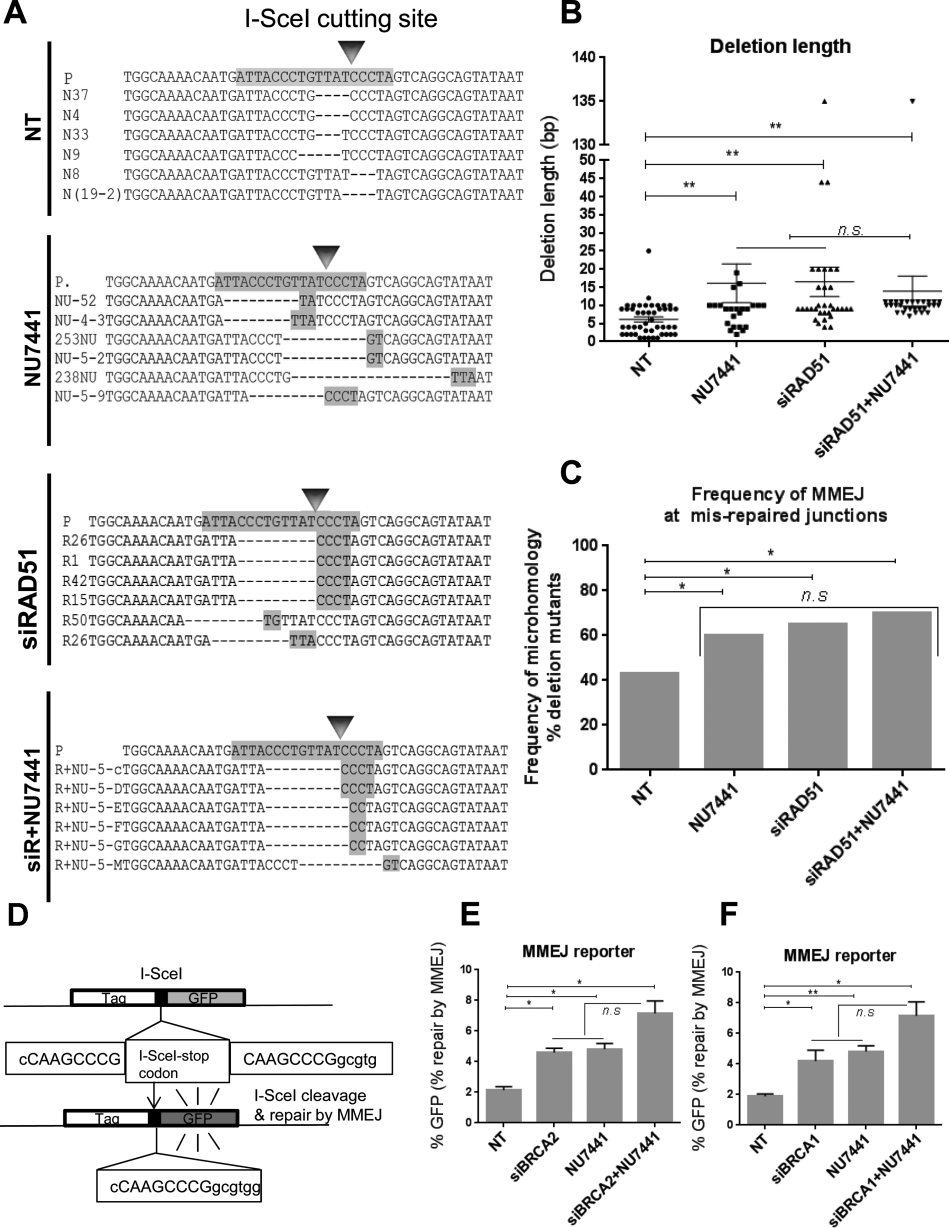
MMEJ-like DSB repair in HR-deficient cells is independent of DNA-PKcs. (**A**) Representative sequence alignments of PCR products obtained from HPRT negative cells treated with NT control siRNA (NT), a DNA-PKcs inhibitor (NU7441), siRNA against RAD51 (siRAD51) or siRNA against RAD51 plus NU7441 (siR+NU7441), from three independent experiments (see also Supplementary Figures S1A, S5-S6). I-SceI recognition sequence and terminal microhomologies at the break sites are highlighted. (**B**) Average length of deletions (bp) in different genetic backgrounds. Each dot represents an independent clone. The lines represent mean and SEM, n.s., not significant, ** *P* < 0.01. (**C**) Frequency of MMEJ at mis-repaired junctions in HPRT deletion mutants isolated from cells treated with NT control siRNA (NT), NU7441, RAD51 siRNA (siRAD51) or RAD51 and NU7441 (siRAD51+NU7441). *P* values calculated by statistical analysis ‘‘difference between proportions’’, *P < 0.05. (**D**) Schematic map of the EJ2-GFP reporter (53) to assess MMEJ efficacy, where the I-SceI cut site is flanked by 8 nucleotide homologous sequences capable of bridging the I-SceI-induced DSB by MMEJ and thus restoring a functional GFP cassette. (**E**) MMEJ repair efficacy of EJ2-GFP reporter cells treated with NT control siRNA (NT), BRCA2 siRNA (siBRCA2), DNA-PKcs inhibitor (NU7441) or BRCA2 and DNA-PKcs co-depleted cells (siBRCA2+NU7441), indicated by the percentage of GFP-positive cells. Error bars show SEM from three independent experiments. *P < 0.05. (**F**) MMEJ repair efficacy of EJ2-GFP reporter cells treated with NT control siRNA (NT), BRCA1 siRNA (siBRCA1), DNA-PKcs inhibitor (NU7441) or BRCA1 siRNA and DNA-PKcs (siBRCA1+NU7441), indicated by the percentage of GFP-positive cells. Error bars show SEM from three independent experiments. *P < 0.05, ** *P* < 0.01.

Although the microhomologies we observed at repair junctions in HR-defective cells were highly suggestive that MMEJ is the key pathway involved, we wanted to test the possible involvement of C-NHEJ. Because C-NHEJ, but not MMEJ, is dependent on DNA-PKcs, we therefore, compared the HPRT mutational profile following treatment of cells with a DNA-PKcs inhibitor (NU7441), or siRNA against RAD51 in the presence and absence of NU7441. This inhibitor has previously been shown to disrupt the C-NHEJ repair pathway (57). We found that treating cells with NU7441 resulted in a significant increase in the deletion length (16 bp, *P* = 0.015) and a significantly increased presence of microhomologies at the break sites (60%, *P*< 0.05) compared to that of the NT background (Figure 3A–C and Supplementary Figure S5). This is consistent with a role for DNA-PKcs in conjunction with Ku in protecting DSB ends and thereby promoting C-NHEJ and counteracting MMEJ (25,58). Further, we found that the RAD51-depleted mutational pattern following DSB induction was largely unaffected by the presence of the DNA-PKcs inhibitor. In particular, similar deletion lengths and use of microhomologies at the HPRT break-sites were observed following RAD51 depletion in the absence or presence of NU7441 (*P* >0.05) (Figure 3A–C, and Supplementary Figure S6). No statistical difference was found between the MMEJ levels in cells treated with NU7441, siRNA against RAD51 or siRNA against RAD51 in combination with NU7441 (Figure 3C). These observations suggested that the DSB mutational signature in the absence of HR is largely independent of DNA-PKcs, a core C-NHEJ factor. To confirm this in another system, we used a well-defined GFP-based MMEJ reporter integrated within a U2OS cell line (EJ2-GFP) (53) (Figure 3D). Knockdown of BRCA2 in this system significantly increased the GFP-positive cells, indicative of MMEJ repair, compared to the NT controls (1.95-fold, *P* = 0.0105) (Figure 3E), confirming that BRCA2 suppresses MMEJ. Also, DNA-PKcs inhibition by NU7441 in this reporter cell line significantly increased the MMEJ repair events by 2.24-fold (*P* = 0.0129) (Figure 3E), consistent with a well-established role for C-NHEJ factors in suppressing MMEJ (42). Further, inhibition of DNA-PKcs by NU7441 in BRCA2-depleted cells led to a slight increase of the GFP-positive cells compared to that of BRCA2-depleted cells (1.62-fold; *P* = 0.0523) or control cells treated with NU7441 (1.4-fold; *P*>0.05) (Figure 3E). When we repeated these experiments using BRCA1 depletion in place of BRCA2 depletion, we obtained very similar results (Figure 3F). These data obtained from the GFP reporter are consistent with those from the HPRT assay; in neither system did treatment with DNA-PKcs inhibitor impair the increase in MMEJ resulting from depletion of an HR protein suggesting that DNA-PKcs is dispensable for the induction of MMEJ in the HR-deficient cells.

Finally, to confirm that NU7441 efficiently inhibits C-NHEJ, we treated the previously described GFP-based C-NHEJ reporter (IRES-TK-EGFP, H1299 cells) (54) (Supplementary Figure S7A) with NU7441 and observed a 93.6% (*P* < 0.0001) fall in C-NHEJ (Supplementary Figure S7B). RAD51 depletion was confirmed by western blot (Supplementary Figure S7C). Collectively these data suggest that MMEJ in HR-impaired cells is independent of DNA-PKcs, a key factor for the C-NHEJ pathway. Also, the GFP-based reporter data suggest that BRCA2 and BRCA1 suppress MMEJ.

### DSB-induced mutation signatures in HR-deficient cells require MMEJ factors

**Figure 4.**
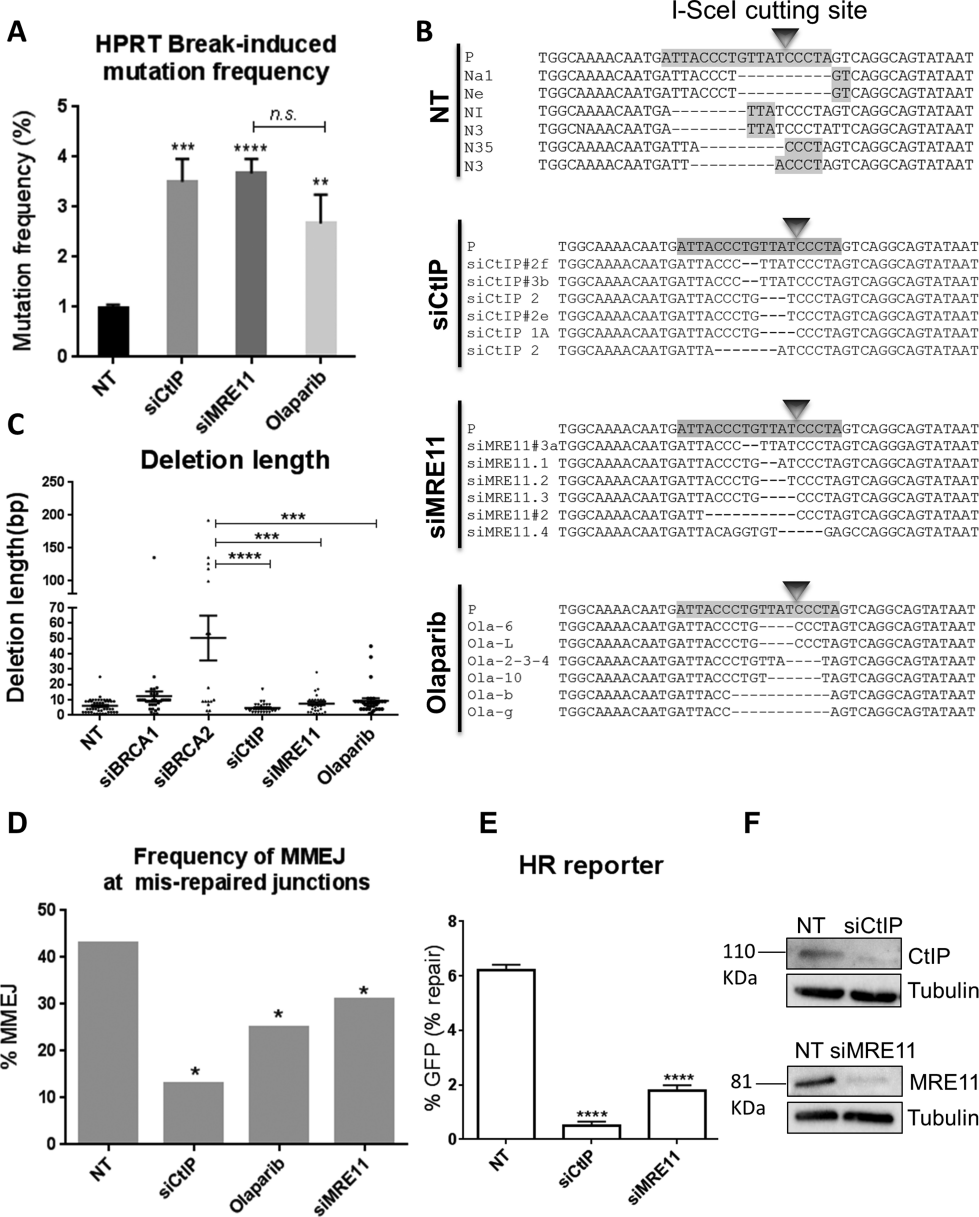
DSB-induced mutation signatures in HR-deficient cells require MMEJ factors. (**A**) Break-induced mutation frequency of HPRT reporter cells treated with NT control siRNA (NT), CtIP siRNA (siCtIP), MRE11 siRNA (siMRE11) or PARP-1 inhibitor (Olaparib). Error bars represent SEM from three independent experiments, n.s., not significant, **P < 0.01, ***P < 0.001, ****P < 0.0001. (**B**) Representative sequence alignments of the HPRT negative PCR products in cells treated with NT control siRNA (NT), CtIP siRNA (siCtIP), MRE11 siRNA (siMRE11) or PARP-1 inhibitor (Olaparib), from three independent experiments (see also Supplementary Figures S1A, S8–10). I-SceI recognition sequence and terminal microhomologies at the break sites are highlighted. (**C**) Average deletion lengths (bp) in different genetic backgrounds. Each dot represents an independent clone. The lines represent mean and SEM, ***P < 0.001, ****P < 0.0001. (**D**) Frequency of MMEJ at mis-repaired junctions in HPRT deletion mutants isolated from cells treated with NT control siRNA (NT) or CtIP siRNA (siCtIP), MRE11 siRNA (siMRE11) or Olaparib. *P* values calculated by statistical analysis ‘‘difference between proportions’’, *P < 0.05. (**E**) HR repair efficacy of DR-GFP reporter cells treated with NT control siRNA (NT), CtIP siRNA (siCtIP), or MRE11 siRNA (siMRE11), indicated by the percentage of GFP-positive cells. Error bars show SEM from three independent experiments. ****P < 0.0001. (**F**) Western blot showing CtIP and MRE11 knockdowns 48 h following siRNA transfection.

The above evidence shows clearly that DSB mis-repaired junctions with microhomologies typical of MMEJ are enhanced when HR is impaired, and that this effect is C-NHEJ-independent. We nevertheless sought direct evidence that MMEJ is indeed the pathway responsible for these mutational signatures. We therefore used the HPRT system to examine the DSB-induced mutational signatures following depletion of essential MMEJ factors. Given that HR and MMEJ share the initial resection step via CtIP and MRE11 (8,59,60), we investigated the impact of depleting these factors on the DSB-induced mutational signature. CtIP and MRE11 were depleted by siRNA-mediated knockdown in the HPRT reporter cells and the mutational frequencies following I-SceI-induced break were quantified. We found that, similar to the depletion of RAD51, SETD2, BRCA2 and BRCA1, depletion of either CtIP or MRE11 resulted in a significant increase in the I-SceI-induced mutation frequency (3.5%; *P* = 0.0007; and 3.6%; *P* < 0.0001, respectively) compared to 1.1% in NT controls (Figure 4A). The mutational frequencies in these backgrounds were slightly but significantly greater than those in BRCA2 depleted cells (*P*<0.05), consistent with roles for CtIP, and MRE11 upstream of BRCA2 during HR. Surprisingly, sequence analysis of HPRT-negative clones derived from CtIP- or MRE11-depleted cells identified a distinct mutational signature compared to that of SETD2-, RAD51-, BRCA2- or BRCA1-depleted cells (Figure 4B, and Supplementary Figures S8 and S9). This signature involved deletions with comparable lengths to those in NT controls (mean 5.6 bp), in contrast to the increased deletion lengths seen in BRCA2-depleted cells (mean 49 bp; *P* < 0.0001, and *P* < 0.001, respectively) (Figures 1C and 4C). Further analysis of the mis-repaired junctions of HPRT-negative clones from these backgrounds identified a significant decrease in the proportion of repair junctions associated with microhomologies in both CtIP- (13%; *P* < 0.05), and MRE11- (31%; *P* < 0.05) depleted cells, compared to NT controls (43% Figure 4D), or to BRCA2 72 (Figure 1D) and BRCA1-depleted cells (65%, Figure 2D). In summary, depletion of CtIP or MRE11, essential MMEJ factors that promote the initiation of resection, significantly reduced the proportion of DSB-induced deletions that were associated with microhomologies, relative to the NT controls. This contrasts sharply with depletion of the HR proteins SETD2, RAD51, BRCA2 or BRCA1, which significantly enhanced the same proportion (Figures 1D and 2D) (51).

To assess the effect of CtIP and MRE11 depletion on HR, we knocked down CtIP and MRE11 in the DR-GFP reporter (U2OS cells) (52) and found that the frequency of HR was significantly reduced by 92% (*P* < 0.0001), and 71% (*P* < 0.0001), respectively (Figure 4E). CtIP and MRE11 depletions were confirmed by western blot (Figure 4F). Together, these findings are in accordance with roles for CtIP and MRE11 in the initiation of resection during both HR and MMEJ.

PARP-1 is also known to play a key role in MMEJ by competing with Ku to bind DSBs, which in turn leads to the recruitment of MRE11 and CtIP to trigger resection (33,61). Consistent with this, Olaparib has recently been shown to decrease the Alt-NHEJ levels in the MMEJ-GFP reporter assay (62). Therefore, we treated the HR-proficient HT1080 cells (HPRT reporter) with a PARP-1 inhibitor, Olaparib (AZD2281) (63), and examined the DSB mutational pattern. The effects of Olaparib were similar to those of CtIP or MRE11 depletion. Thus Olaparib significantly increased the mutation frequency (2.6%; *P* = 0.0073; Figure 4A) compared to the 1.1% in NT controls, generating deletion lengths (mean 7 bp) comparable to the controls (mean 5.6 bp; *P* > 0.05; Figure 4B and C), and significantly shorter than deletion lengths in BRCA2-depleted HPRT mutants (mean 49 bp; *P* = 0.0005). Similarly, HPRT mutants arising following DSB induction in cells treated with Olaparib showed a significant reduction in the proportion of deletion junctions associated with microhomologies (25%; *P* <0.05) relative to NT controls (57%) (Figure 4B, D and Supplementary Figure S10), Collectively, these results suggest that microhomologies at DSB mis-repaired junctions in the HPRT reporter do indeed originate from the MMEJ repair machinery.

To confirm that the MMEJ-like signature in HR-compromised cells arises from MMEJ, we aimed to see whether knocking down a core MMEJ factor could abolish the MMEJ signature in these cells. We therefore, co-depleted BRCA1 and MRE11 in the HPRT system and monitored the DSB mutational signature. Simultaneous knockdown of BRCA1 and MRE11 resulted in a significantly increased mutation frequency of 3.4% compared to that of the NT background (1.1%, *P* = 0.0013) (Supplementary Figure S11A). This increased frequency of HPRT loss is consistent with the roles for BRCA1 and MRE11 in promoting DSB repair (14,15). Further, the MMEJ signature was absent in cells co-depleted for BRCA1 and MRE11. This was associated with deletion lengths comparable to that of NT cells (mean 5.6 bp), and a significantly reduced proportion of MMEJ (27%) compared to that of NT cells (43%, *P* < 0.05) (see Supplementary Figures S11 and S12). Together these data confirm that the MMEJ-like signature in the absence of HR factors is arising from the MMEJ pathway.

### RPA suppresses MMEJ

**Figure 5.**
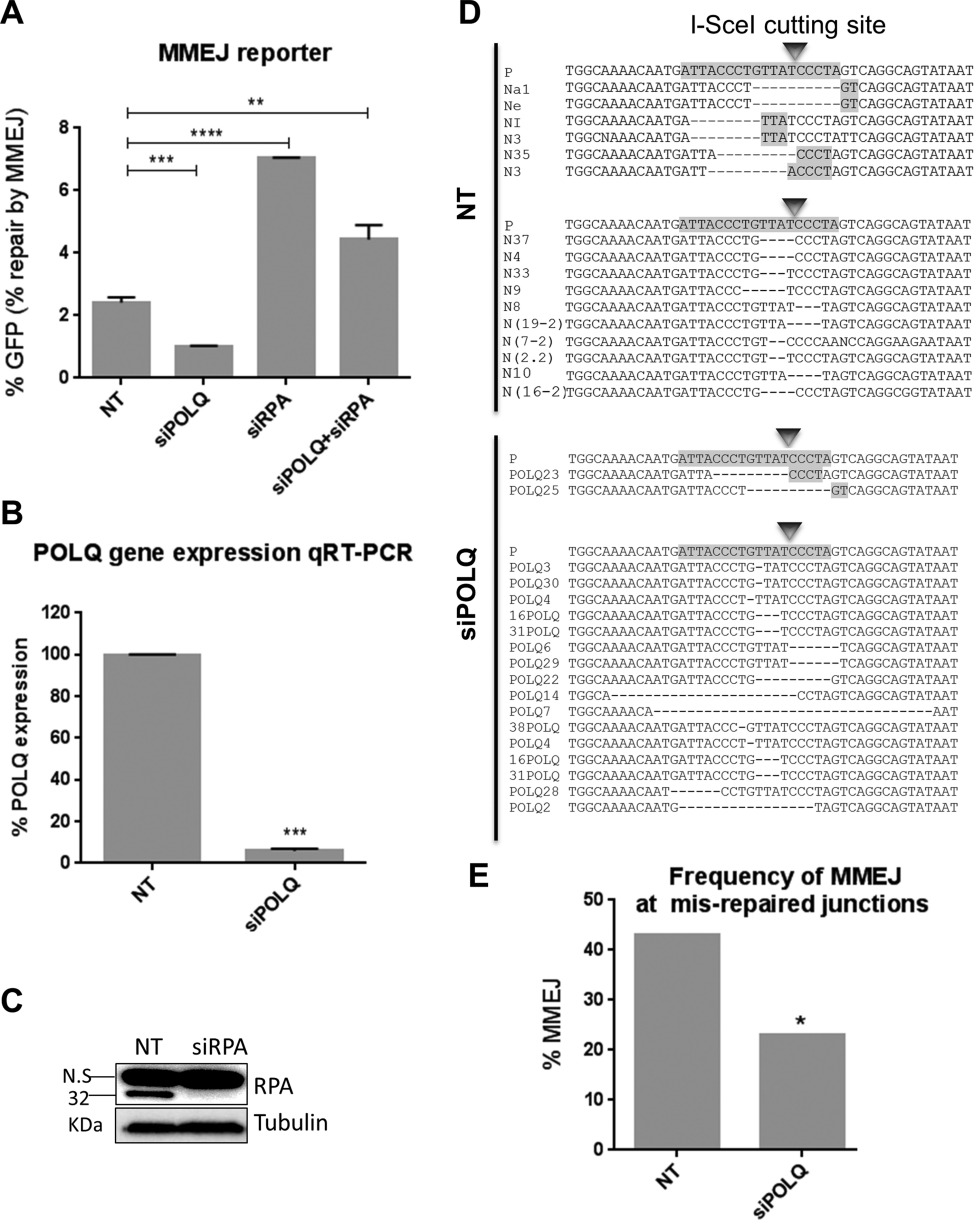
RPA suppresses MMEJ. (**A**) MMEJ repair efficacy of EJ2-GFP reporter cells treated with NT control siRNA (NT), POLQ siRNA (siPOLQ), RPA siRNA (siRPA) and POLQ plus RPA (siPOLQ+siRPA) indicated by the percentage of GFP-positive cells. Error bars show SEM from three independent experiments. **P < 0.01, ***P < 0.001, ****P < 0.0001. (**B**) POLQ gene expression measured by RT-qPCR using the primers indicated in Materials and Methods. (**C**) Western blot showing RPA knockdown 48 h following siRNA transfection. (**D**) Representative sequence alignments of the PCR products (see Supplementary Figure S13) in NT control (NT), and POLQ-depleted (siPOLQ) cells. I-SceI recognition sequence and terminal microhomologies at the break sites are highlighted. (**E**) Frequency of MMEJ at mis-repaired junctions in HPRT deletion mutants isolated from cells treated with NT control siRNA (NT) or POLQ siRNA (siPOLQ). *P* values calculated by statistical analysis ‘‘difference between proportions’’, * *P* < 0.05.

RPA is a heterotrimeric ssDNA binding protein with roles in DNA replication, recombination and DSB repair, including HR. RPA displacement by RAD51 is a critical step in HR (18). In this regard, it has been previously shown in *S. cerevisiae* that RPA antagonizes MMEJ (45). Therefore, to test a possible role for RPA in repressing MMEJ repair in human cells, we used the GFP-based reporter for MMEJ repair (53) (Figure 3D). RPA knockdown (Figure 5C) resulted in significantly enhanced MMEJ levels compared to the NT controls (2.9-fold; *P* < 0.0001) (Figure 5A), suggesting that human RPA also suppresses MMEJ. POLQ has recently been identified as an MMEJ-promoting factor in mammalian cells (46,47). Thus siRNA knockdown of POLQ was used as a control for the GFP-based MMEJ reporter, which led to 94% reduction in *POLQ* gene expression levels, as evaluated by qRT-PCR (Figure 5B). This was found to significantly reduce MMEJ repair by 58% (*P* = 0.0112), consistent with its role in the mammalian MMEJ pathway (Figure 5A). Further, co-depletion of RPA and POLQ in the GFP-based MMEJ reporter led to significantly reduced levels of MMEJ compared to that of RPA-depleted cells (*P* = 0.0007). Surprisingly, however, MMEJ levels in cells co-depleted for POLQ and RPA were still significantly greater than NT controls (*P* = 0.0022) (Figure 5A), suggestive of the possible existence of POLQ-dependent and POLQ-independent MMEJ pathways being suppressed by RPA. Finally, to confirm the role for POLQ in promoting MMEJ in the HPRT assay, we depleted POLQ in this system and monitored the DSB mutational pattern. Analysis of the mutational signature in cells treated with siRNA against POLQ showed a significantly decreased MMEJ levels compared to that of the NT background, (Figure 5D and E and Supplementary Figure S13). These results resemble our findings in the MMEJ-GFP assay, confirming that the HPRT and the GFP reporter assays measure the same process.

## DISCUSSION

**Figure 6.**
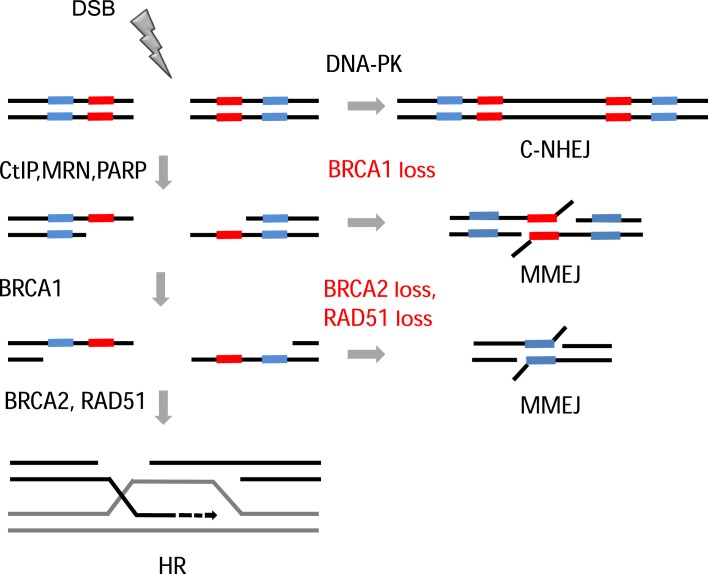
Model. DSBs are mainly repaired by C-NHEJ, however upon resection initiation by CtIP and MRN complex repair occurs via HR pathway. If HR is disrupted after initiation of resection, regions of microhomology on either side of the break will anneal, resulting in repair via the mutagenic MMEJ pathway.

Mutations arising from aberrant DSB repair are deleterious for cells and can give rise to genomic instability, a hallmark of cancer (64). Yet how DSB-induced chromosomal rearrangements are suppressed is poorly characterized. Further, there is little mechanistic insight into the relationship between disrupting repair pathways and the mechanisms of DSB mis-repair. Here, using an I-SceI-cleavable reporter assay based on the human endogenous *HPRT* gene (50,51), together with an array of I-SceI-cleavable GFP-based reporter assays (52–54), in different cancer cell lines we have established a role for HR factors in suppressing mutagenic MMEJ following DSB resection. In this context, we consider MMEJ to arise as the default pathway following HR inactivation rather than being actively suppressed by HR (see model in Figure 6). Following on from our initial observations in RAD51- and SETD2-depleted cells (51), this study confirms that depletion of other HR factors (BRCA2, BRCA1 and RPA) also results in significantly elevated levels of a common DSB mutational signature in which DSB-induced deletions are associated with microhomologies of 2–6 bp at the break junctions. Our observations are in alignment with the recent studies in human cells indicating that chromosomal rearrangement junctions formed by MMEJ contain 2–6 bp of microhomology regions (38). Further, our data also indicate that while failed HR leads to an increase in MMEJ-induced deletions, surprisingly no effect was observed on insertions, suggesting that these arise through other mutagenic NHEJ events in this context. In this respect, insertions were still observed, albeit at low levels, following inhibition of DNA-PKcs or following knockdown or inhibition of MMEJ factors, suggesting these insertions arise through other end-joining mechanisms. However, this needs to be investigated in more detail.

Because the junctional microhomologies can potentially represent DSB mutational signatures of either C-NHEJ or MMEJ pathways (65,66), it is important to know which of these pathways is involved. However the abrogation of these signatures following depletion or inhibition of CtIP, MRE11, PARP-1, all of which facilitate MMEJ, provides strong genetic evidence that the MMEJ pathway is responsible. The observation that MMEJ levels are decreased following PARP-1 inhibition with Olaparib in the HPRT system, is in agreement with a recent study in which a similar finding was found using a GFP-based reporter assay (62).

Additionally, the persistence of junctional microhomologies in HR-depleted cells treated with DNA-PKcs inhibitor excludes the possibility that C-NHEJ is required. These conclusions are further supported by the significantly greater levels of MMEJ events detected by a GFP-based reporter following depletion of HR factors, even when DNA-PKcs is inhibited (Figure 3E and F). Our findings therefore indicate that, following DSB induction, HR proteins preserve genomic integrity by suppressing mutations arising through the MMEJ pathway. MMEJ can be considered an important DSB repair pathway in its own right. However our data suggest that its levels can be increased as a result of disrupted HR. In this respect, we know MMEJ has been proposed to be higher during S/G2 (59), which may arise through incomplete HR. For example, one-ended breaks arising from replication collapse may give rise to both MMEJ and HR.

While the role of CtIP and the MRN complex in resection initiation is well established (67), the exact role of BRCA1 in resection remains uncertain (68–70). It has recently been reported that, although the BRCA1-CtIP interaction is not crucial for DNA end-resection, it enhances the speed of CtIP-mediated resection (17). It is also known that CtIP and MRE11 are required for both HR and MMEJ repair pathways by triggering resection (59). Differing results regarding the contribution of BRCA1 in MMEJ have been reported. Thus, while one recent study in MEFs found that BRCA1 facilitates MMEJ at uncapped telomeres (49), work in DT40 (chicken) B cells suggested that MMEJ is not influenced by BRCA1 (70). Various studies of plasmid-based (exogenous) DSB repair substrates in human cells, however, provide evidence that BRCA1 represses Alt-NHEJ events by promoting the fidelity of NHEJ. (48,71–73). Here, using an endogenous HPRT assay together with GFP reporter assays in different human cell lines, we identified a DSB-induced mutational signature for BRCA1-depleted cells, which is distinct from that of CtIP-depleted or MRE11-depleted cells. The significantly greater HPRT deletion lengths in BRCA1-depleted cells compared to those of CtIP or MRE11-depleted cells supports the notion that BRCA1 operates downstream of CtIP and MRE11. Further, the significantly smaller HPRT deletion lengths in BRCA1-depleted cells compared to those of BRCA2-depleted cells, suggests that BRCA1 functions downstream of CtIP and MRE11 but upstream of BRCA2 during HR to promote genome stability by facilitating resection and thereby suppressing the mutagenic MMEJ. Moreover, the significantly increased levels of MMEJ, detected by both the HPRT and GFP assays, following BRCA1 depletion suggest that BRCA1 suppresses MMEJ repair. This is in agreement with previous studies using plasmid-based reporter systems (48,71).

POLQ has recently been identified as a polymerase that contributes to mammalian MMEJ by promoting DNA end joining and microhomology annealing (37,38). Further, recent studies have suggested that cancer cells with defective HR are dependent on POLQ-mediated MMEJ repair (46,47). Moreover, POLQ is upregulated in a range of human cancers, and indicates a poor clinical prognosis, especially in breast tumours (74–76). In agreement with these reports, our observations support a role for POLQ in facilitating MMEJ in human cancer cells. Also RPA has an established role in repressing MMEJ in *S. cerevisiae* by removing DNA secondary structures, which prevent RAD51 loading and therefore HR (45,77). Consistent with this, we demonstrate a striking role for RPA in suppressing mutagenic MMEJ in human cancer cells. This could reflect roles for RPA in early HR, thereby suppressing MMEJ and/or in binding ssDNA, thereby preventing spontaneous MMEJ, as previously proposed in *S. cerevisiae* (45). Our data, however, also identify dramatically increased MMEJ levels in cells following simultaneous depletion of POLQ and RPA. This is indicative of a role for RPA in suppressing MMEJ independently of POLQ, which could be explained by the suggested involvement of POLQ in a subset of Alt-NHEJ repair pathways specifically leading to insertions (78,79). Our results also suggest that there might be POLQ-dependent and POLQ-independent MMEJ pathways in human cancer cells, both of which are suppressed by RPA. Our data do not, however, exclude the possibility that the slight residual activity of POLQ following its siRNA-mediated knockdown, may contribute to the great MMEJ levels in cells co-depleted for RPA and POLQ. In this regard, a more in-depth analysis is required.

While our data show that MMEJ is enhanced when HR is impaired, the nature of the corresponding decrease in other forms of mutagenic NHEJ is less clear and could indicate inhibition of indel-forming C-NHEJ or other Alt-NHEJ events that are not dependent on junctional microhomologies (6). Further analyses are required to identify which DSB repair pathways are affected.

Our combined observations support a model (Figure 6) in which DSB repair in HR-proficient cells, results in a mutational signature with a particular balance between deletions with and without associated microhomologies. When HR is impaired, however, this balance is channeled toward deletions associated with microhomologies as a result of enhanced MMEJ. Mechanistically, we propose that, following DSB induction and initiation of resection, the repair pathway choice can switch from HR to MMEJ, depending on the availability of HR downstream proteins. In the absence of HR factors, HR is blocked, and the resected ends are poor substrates for C-NHEJ. As a result, microhomologous sequences present on ssDNA either side of the resected ends anneal via the mutagenic MMEJ repair mechanism leading to microhomology-mediated deletions. Also consistent with our findings, a recent study using MEFs found increased translocation levels following Rad54 deletion (44). RAD54 is a key HR factor promoting recombination through interactions with RAD51 (80). Although there is no sequence-based evidence for the use of microhomologies in generating translocation junctions, translocations were largely PARP-1- and Lig 3-dependent suggesting that they had arisen via the MMEJ pathway (39,81).

Mutations in DNA DSB repair genes are common features in different human cancer types. Among the DSB repair pathways, MMEJ is necessarily mutagenic and is frequently associated with genomic rearrangements (39,82). Importantly, high-resolution sequencing studies have identified microhomologies as a prevalent mutational signature at rearrangement breakpoints in several cancer types including breast, colorectal and prostate adenocarcinomas (83,84). Our observations therefore suggest that the microhomology mutational signature in different types of malignancies may arise from failed HR at a stage after the initiation of resection. This mechanistic understanding of the interplay between HR and MMEJ repair pathways could be exploited to develop therapies for cancer patients deficient in these DSB repair pathways.

## Supplementary Material

SUPPLEMENTARY DATA
