# Supplementary material for: A role for human homologous recombination factors in suppressing microhomology-mediated end joining
Source: Nucleic Acids Res. 2016 Apr 29;44(12):5743–57. doi: 10.1093/nar/gkw326 (PMC4937322; doi:10.1093/nar/gkw326)
Supplement: SUPPLEMENTARY DATA [file supp_44_12_5743__index.html]

A role for human homologous recombination factors in suppressing microhomology-mediated end joining — SUPPLEMENTARY DATA 

# A role for human homologous recombination factors in suppressing microhomology-mediated end joining

## SUPPLEMENTARY DATA

- SUPPLEMENTARY DATA
- SUPPLEMENTARY DATA
