# Supplementary material for: A role for human homologous recombination factors in suppressing microhomology-mediated end joining
Source: Nucleic Acids Res. 2016 Apr 29;44(12):5743–57. doi: 10.1093/nar/gkw326 (PMC4937322; doi:10.1093/nar/gkw326)
Supplement: SUPPLEMENTARY DATA [file supp_gkw326_nar-03361-d-2015-File008.docx]

**Ahrabi et al Supplementary data**

**Legends to Supplementary Figures**

**Fig.S1. HPRT reporter**

(A) Schematic of the HPRT assay.

(B) Temporal outline of the HPRT assay.

(C) Stained HPRT negative single cell colonies in the absence and presence of I-SceI-induced damage.

(D) Calculations for the HPRT mutation frequency.

Cells grown for the plating efficiencies were kept in non-selective medium, while cells grown for the mutation frequencies were kept in 6-TG medium.

(E) Cartoon illustrating the formation of the microhomologies at the break site following a break. I-SceI recognition site is shown in bold and microhomologies are highlighted. Annealing of microhomologies results in nucleotide deletions shown in bold black.

**Fig.S2. Sequence alignments of HPRT negative cells obtained from non-targeting (NT) control background**

Results from three independent experiments are shown in Figure S2A. The numbers in the brackets indicate the experiment each sequence has been derived from. The results from two further independent experiments are shown in Figures S2B (expt4) and S2C (expt 5), respectively to confirm the reproducibility of the assay. HPRT deletion mutants harbouring microhomologies at the breakpoint junctions (consistent with MMEJ) are shown on the top. HPRT deletion mutants with no microhomologies at breakpoint junctions (consistent with other mutagenic NHEJ) are shown on the bottom. Each line represents an individual HPRT negative clone. The parental uncut sequence (P) is shown on the top with the I-SceI recognition site highlighted in gray and the I-SceI cut site indicated with an arrow. Microhomologies are highlighted in gray adjacent to deleted nucleotides. Average deletion lengths and the proportion of MMEJ events relative to other mutagenic NHEJ events are shown. Total of 30 individual cell colonies were isolated from each experiment for the subsequent statistical analysis.

**Fig.S3.Sequence alignments of HPRT negative cells obtained from BRCA2-depleted background**

HPRT deletion mutants harbouring microhomologies at the breakpoint junctions (consistent with MMEJ) are shown on the top. HPRT deletion mutants with no microhomologies at breakpoint junctions (consistent with other mutagenic NHEJ) are shown on the bottom. Each line represents an individual HPRT negative clone. The parental uncut sequence (P) is shown on the top with the I-SceI recognition site highlighted in gray and the I-SceI cut site indicated with an arrow. Microhomologies are highlighted in gray adjacent to deleted nucleotides. Total of 30 individual cell colonies were isolated, from three independent experiments for the purpose of the corresponding statistical analyses.

**Fig.S4. Sequence alignments of HPRT negative cells obtained from BRCA1-depleted background**

HPRT deletion mutants harbouring microhomologies at the breakpoint junctions (consistent with MMEJ) are shown on the top. HPRT deletion mutants with no microhomologies at breakpoint junctions (consistent with other mutagenic NHEJ) are shown on the bottom. Each line represents an individual HPRT negative clone. The parental uncut sequence (P) is shown on the top with the I-SceI recognition site highlighted in gray and the I-SceI cut site indicated with an arrow. Microhomologies are highlighted in gray adjacent to deleted nucleotides. Total of 30 individual cell colonies were isolated, from three independent experiments for the purpose of the corresponding statistical analyses.

**Fig.S5. Sequence alignments of HPRT negative cells obtained from cells treated the DNA-PKcs inhibitor NU7441**

HPRT deletion mutants harbouring microhomologies at the breakpoint junctions (consistent with MMEJ) are shown on the top. HPRT deletion mutants with no microhomologies at breakpoint junctions (consistent with other mutagenic NHEJ) are shown on the bottom. Each line represents an individual HPRT negative clone. The parental uncut sequence (P) is shown on the top with the I-SceI recognition site highlighted in gray and the I-SceI cut site indicated with an arrow. Microhomologies are highlighted in gray adjacent to deleted nucleotides. Total of 30 individual cell colonies were isolated, from three independent experiments for the purpose of the corresponding statistical analyses.

**Fig.S6. Sequence alignments of HPRT negative cells obtained from cells simultaneously treated with siRNA against RAD51 and NU7441** HPRT deletion mutants harbouring microhomologies at the breakpoint junctions (consistent with MMEJ) are shown on the top. HPRT deletion mutants with no microhomologies at breakpoint junctions (consistent with other mutagenic NHEJ) are shown on the bottom. Each line represents an individual HPRT negative clone. The parental uncut sequence (P) is shown on the top with the I-SceI recognition site highlighted in gray and the I-SceI cut site indicated with an arrow. Microhomologies are highlighted in gray adjacent to deleted nucleotides. Total of 30 individual cell colonies were isolated, from three independent experiments for the purpose of the corresponding statistical analyses.

**Fig.S7. GFP reporter for C-NHEJ**

(A) Schematic map of the IRES-TK-EGFP reporter to assess C-NHEJ efficacy

(B) C-NHEJ repair efficacy of IRES-TK-EGFP reporter cells treated with DMSO (control), and DNA-PKcs inhibitor (NU7441) indicated by the percentage of GFP-positive cells. Error bars show SEM from three independent experiments. **** P < 0.0001.

(C) Western blot, showing RAD51 knockdown 48 hours following siRNA transfection in HT1080 cells.

**Fig.S8. Sequence alignments of HPRT negative cells obtained from CtIP-depleted background**

HPRT deletion mutants harbouring microhomologies at the breakpoint junctions (consistent with MMEJ) are shown on the top. HPRT deletion mutants with no microhomologies at breakpoint junctions (consistent with other mutagenic NHEJ) are shown on the bottom. Each line represents an individual HPRT negative clone. The parental uncut sequence (P) is shown on the top with the I-SceI recognition site highlighted in gray and the I-SceI cut site indicated with an arrow. Microhomologies are highlighted in gray adjacent to deleted nucleotides. Total of 30 individual cell colonies were isolated, from three independent experiments for the purpose of the corresponding statistical analyses.

**Fig.S9. Sequence alignments of HPRT negative cells obtained from MRE11-depleted background**

HPRT deletion mutants harbouring microhomologies at the breakpoint junctions (consistent with MMEJ) are shown on the top. HPRT deletion mutants with no microhomologies at breakpoint junctions (consistent with other mutagenic NHEJ) are shown on the bottom. Each line represents an individual HPRT negative clone. The parental uncut sequence (P) is shown on the top with the I-SceI recognition site highlighted in gray and the I-SceI cut site indicated with an arrow. Microhomologies are highlighted in gray adjacent to deleted nucleotides. Total of 30 individual cell colonies were isolated, from three independent experiments for the purpose of the corresponding statistical analyses.

**Fig.S10. Sequence alignments of HPRT negative cells obtained from cells treated with Olaparib (a PARP-1 inhibitor)**

HPRT deletion mutants harbouring microhomologies at the breakpoint junctions (consistent with MMEJ) are shown on the top. HPRT deletion mutants with no microhomologies at breakpoint junctions (consistent with other mutagenic NHEJ) are shown on the bottom. Each line represents an individual HPRT negative clone. The parental uncut sequence (P) is shown on the top with the I-SceI recognition site highlighted in gray and the I-SceI cut site indicated with an arrow. Microhomologies are highlighted in gray adjacent to deleted nucleotides. Total of 30 individual cell colonies were isolated, from three independent experiments for the purpose of the corresponding statistical analyses.

**Fig.S11. DSB-induced mutation signatures in cells co-depleted for BRCA1 and MRE11**

(A) Break-induced mutation frequency of HPRT reporter cells treated with NT control siRNA (NT), or co-depleted for BRCA1 and MRE11 (siBRCA1+siMRE11). Error bars represent SEM from three independent experiments, n.s., not significant, **P < 0.01, ***P < 0.001, ****P < 0.0001.

(B) Representative sequence alignments of the HPRT negative PCR products in cells treated with NT control siRNA (NT), or co-depleted for BRCA1 and MRE11(siBRCA1+siMRE11), respectively from three independent experiments. Terminal microhomologies at the break sites are highlighted. For the full set of sequences see Supplementary Figure 12S.

(C) Average deletion lengths (bp) in different genetic backgrounds. Each dot represents an independent clone. The lines represent mean and SEM, ***P < 0.001, ****P < 0.0001.

(D) Frequency of MMEJ at mis-repaired junctions in HPRT deletion mutants isolated from cells treated with NT control siRNA or co-depleted for BRCA1 and MRE11(siBRCA1+siMRE11), P values calculated by statistical analysis ‘‘difference between proportions’’, *P < 0.05.

**Fig.S12. Sequence alignments of HPRT negative cells obtained from cells co-depleted for BRCA1 and MRE11 (siBRCA1+siMRE11)**

HPRT deletion mutants harbouring microhomologies at the breakpoint junctions (consistent with MMEJ) are shown on the top. HPRT deletion mutants with no microhomologies at breakpoint junctions (consistent with other mutagenic NHEJ) are shown on the bottom. Each line represents an individual HPRT negative clone. The parental uncut sequence (P) is shown on the top with the I-SceI recognition site highlighted in gray and the I-SceI cut site indicated with an arrow. Microhomologies are highlighted in gray adjacent to deleted nucleotides. A total of 30 individual cell colonies were isolated, from three independent experiments for the purpose of the corresponding statistical analyses.

**Fig.S13. Sequence alignments of HPRT negative cells obtained from cells treated with siRNA against POLQ**

HPRT deletion mutants harbouring microhomologies at the breakpoint junctions (consistent with MMEJ) are shown on the top. HPRT deletion mutants with no microhomologies at breakpoint junctions (consistent with other mutagenic NHEJ) are shown on the bottom. Each line represents an individual HPRT negative clone. The parental uncut sequence (P) is shown on the top with the I-SceI recognition site highlighted in gray and the I-SceI cut site indicated with an arrow. Microhomologies are highlighted in gray adjacent to deleted nucleotides. A total of 30 individual cell colonies were isolated, from three independent experiments for the purpose of the corresponding statistical analyses.

**Fig.S14. Analysis of breakpoint junctions harboring insertions in different genetic backgrounds.**

(A) Frequencies of insertions at the DSB junctions in different genetic backgrounds.

(B) Sequence alignments of the inserted sequences at the DSB junctions in different genetic backgrounds.

(C) Sizes and origins of the inserted sequences at the DSB junctions in cells with different genetic backgrounds.
