# Supplementary figures and images for: A role for human homologous recombination factors in suppressing microhomology-mediated end joining
Source: Nucleic Acids Res. 2016 Apr 29;44(12):5743–57. doi: 10.1093/nar/gkw326 (PMC4937322; doi:10.1093/nar/gkw326)

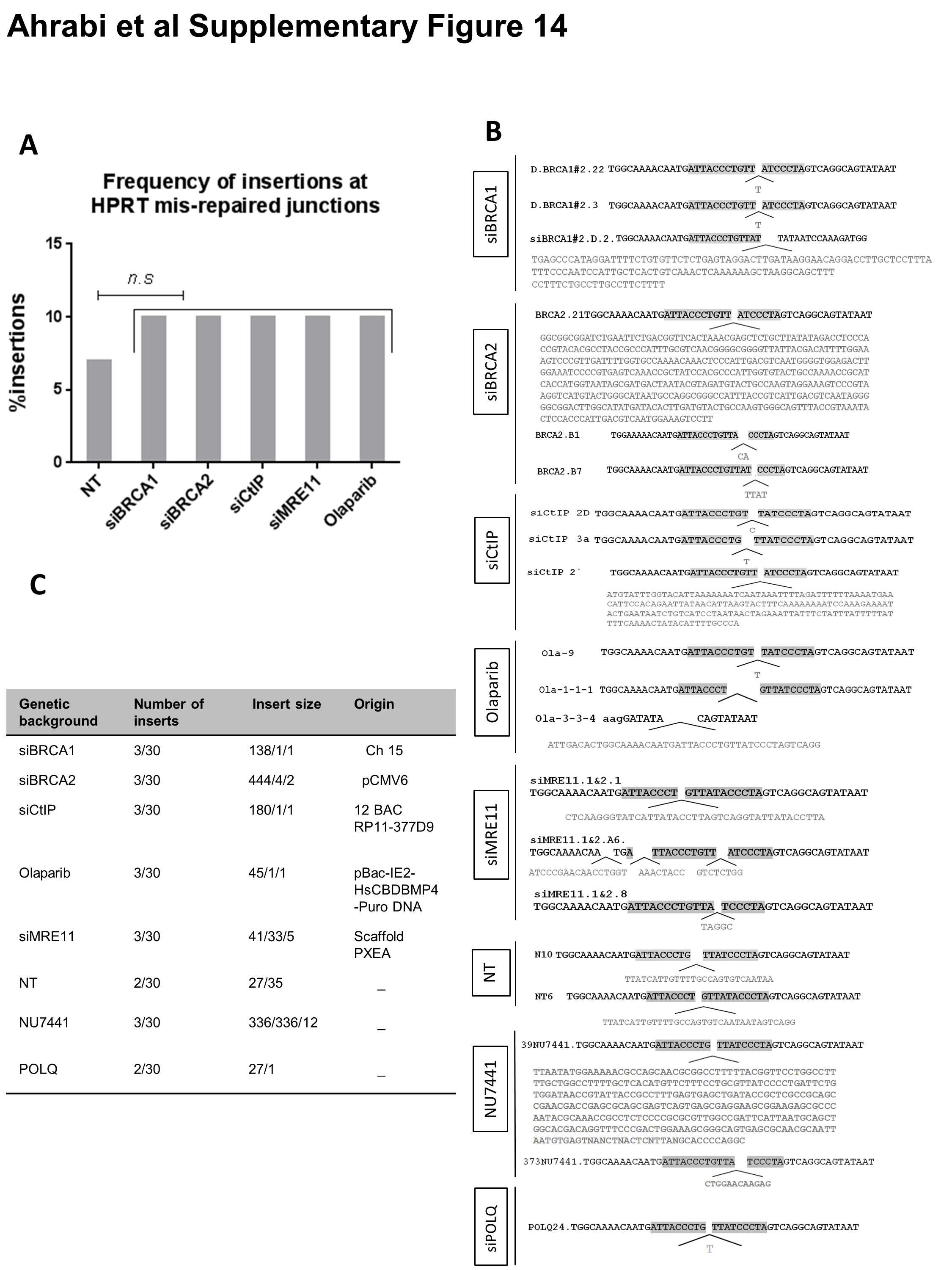

Supplement: SUPPLEMENTARY DATA [file supp_gkw326_nar-03361-d-2015-File009.zip › FigS16.TIF]

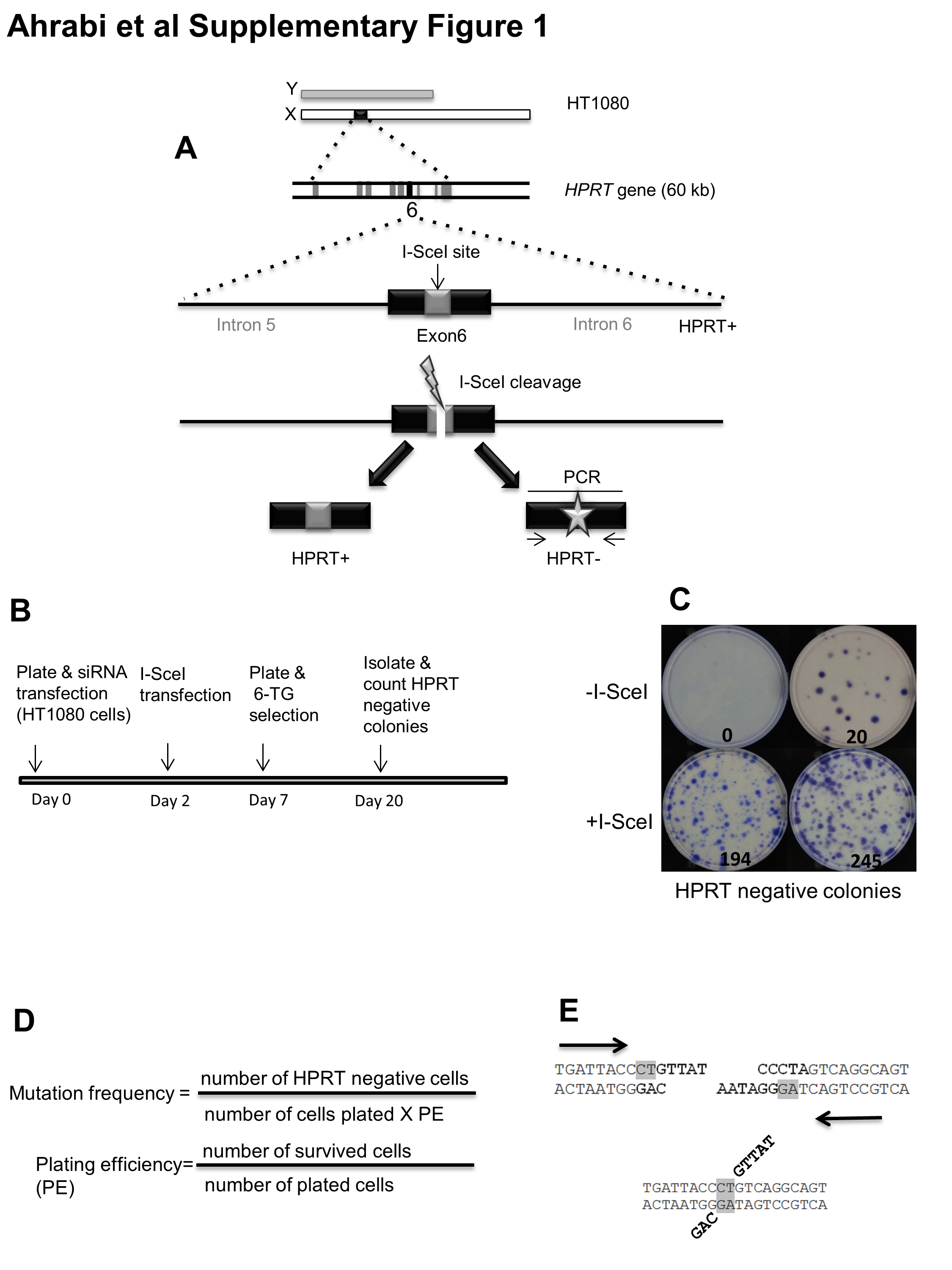

Supplement: SUPPLEMENTARY DATA [file supp_gkw326_nar-03361-d-2015-File009.zip › FigS1.TIF]

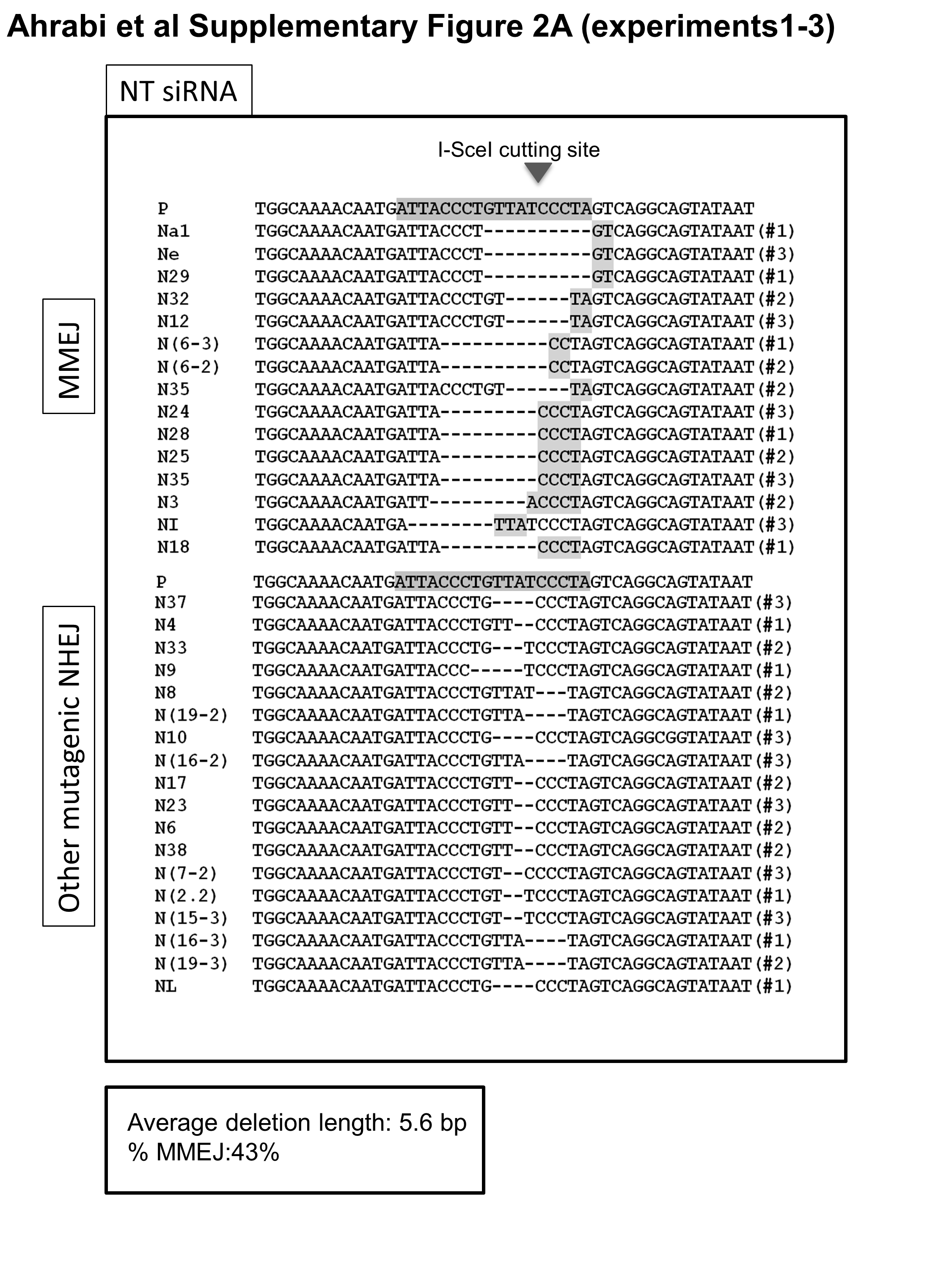

Supplement: SUPPLEMENTARY DATA [file supp_gkw326_nar-03361-d-2015-File009.zip › FigS2.TIF]

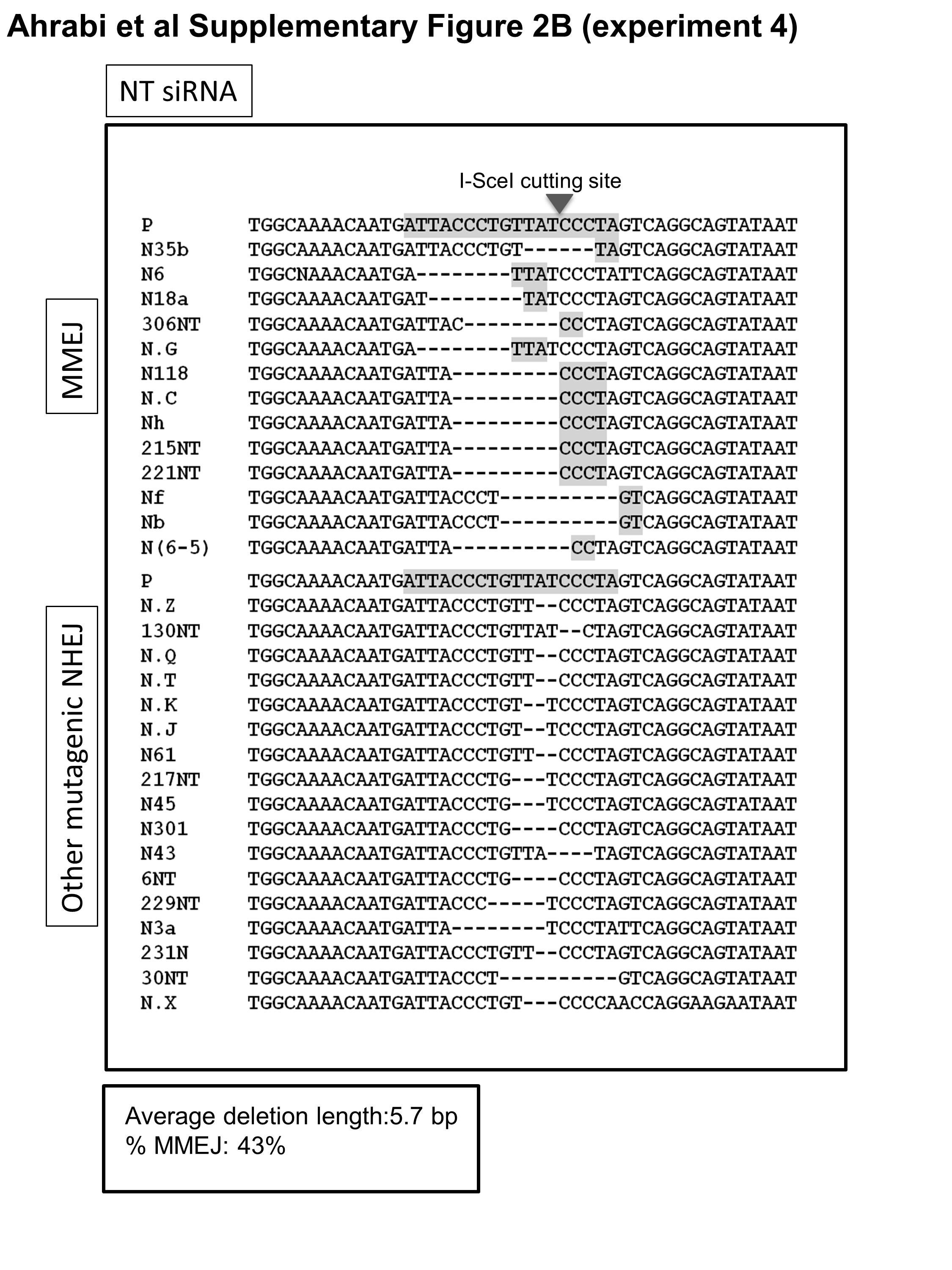

Supplement: SUPPLEMENTARY DATA [file supp_gkw326_nar-03361-d-2015-File009.zip › FigS3.TIF]

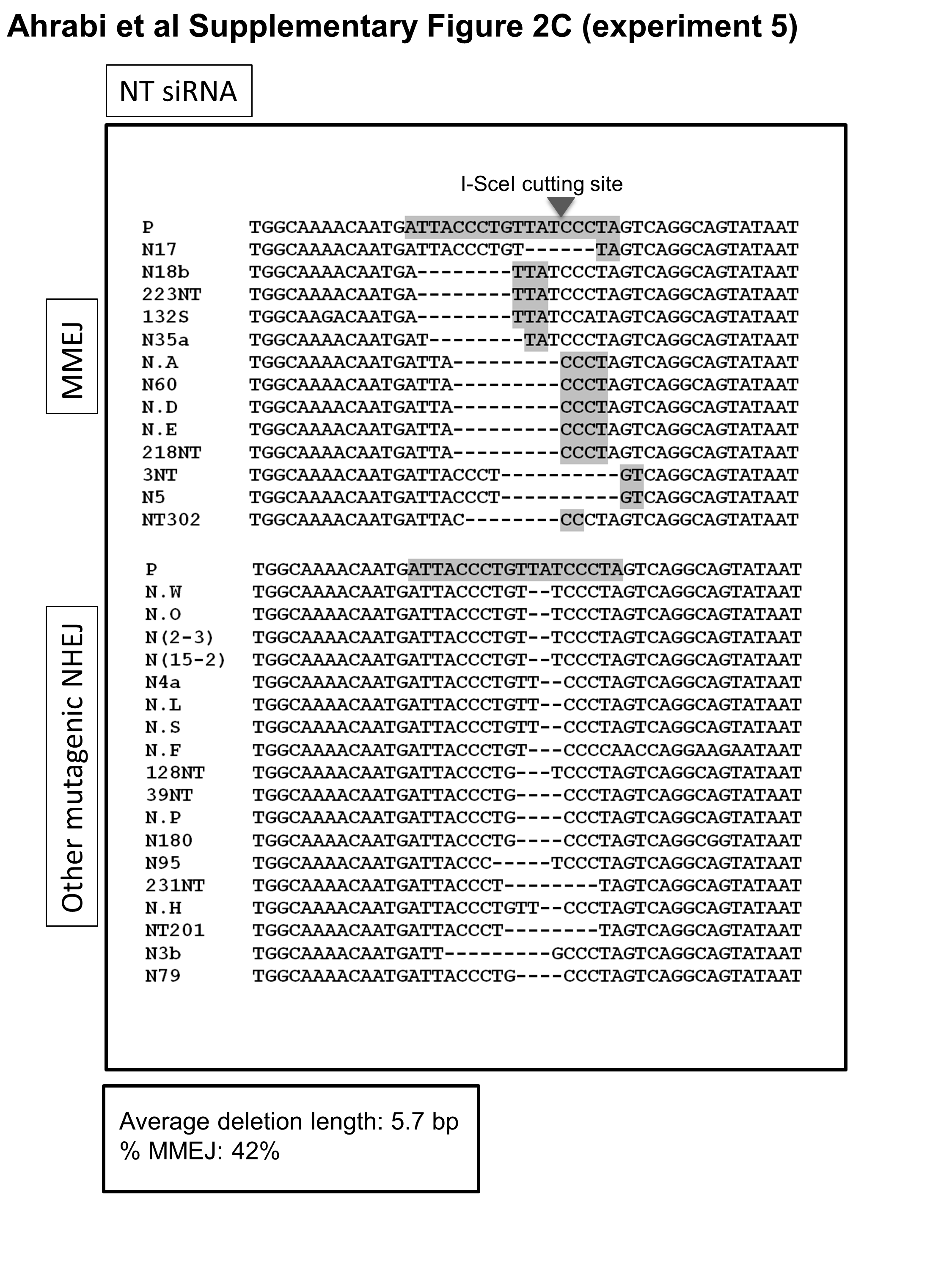

Supplement: SUPPLEMENTARY DATA [file supp_gkw326_nar-03361-d-2015-File009.zip › FigS4.TIF]

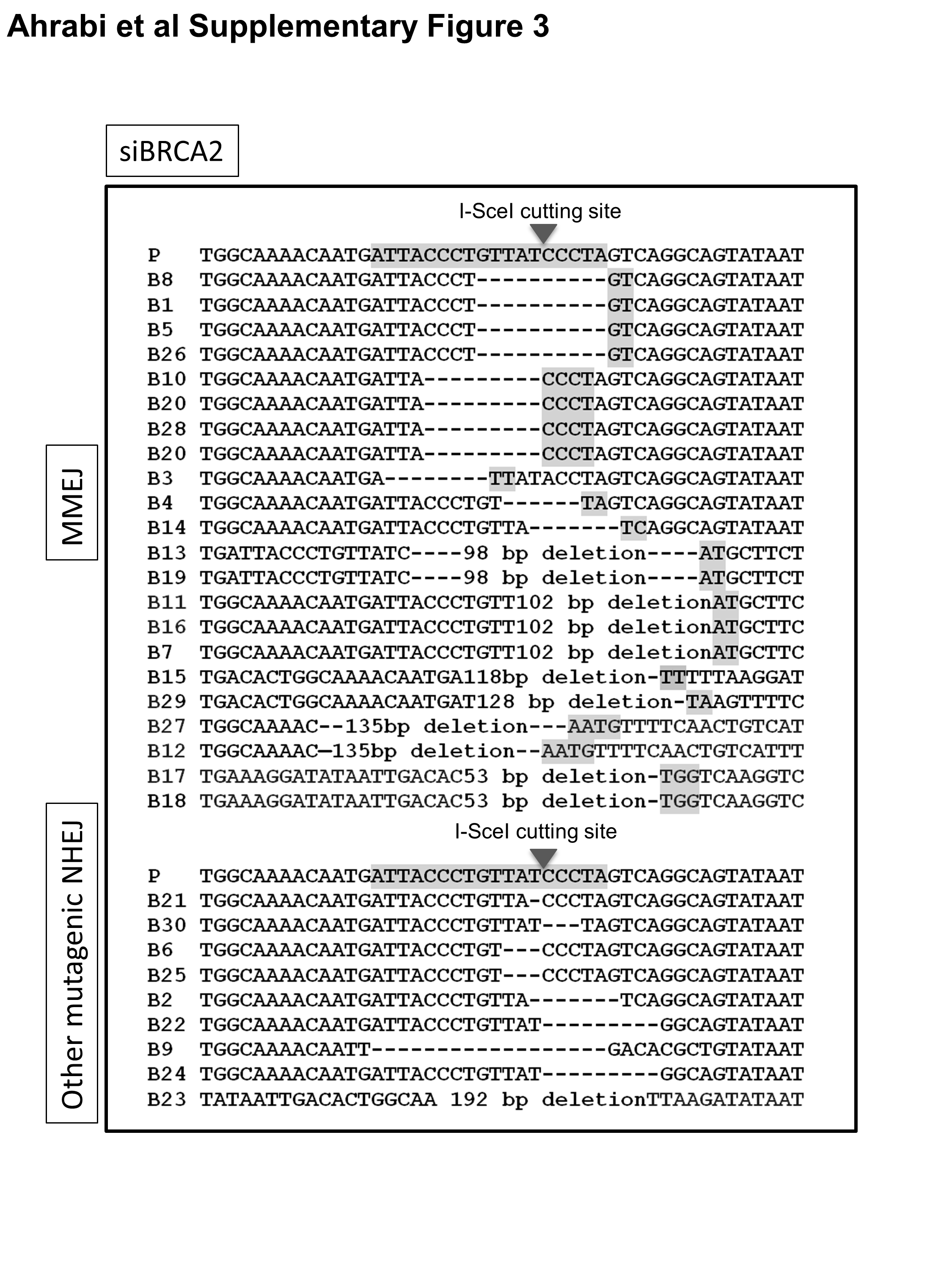

Supplement: SUPPLEMENTARY DATA [file supp_gkw326_nar-03361-d-2015-File009.zip › FigS5.TIF]

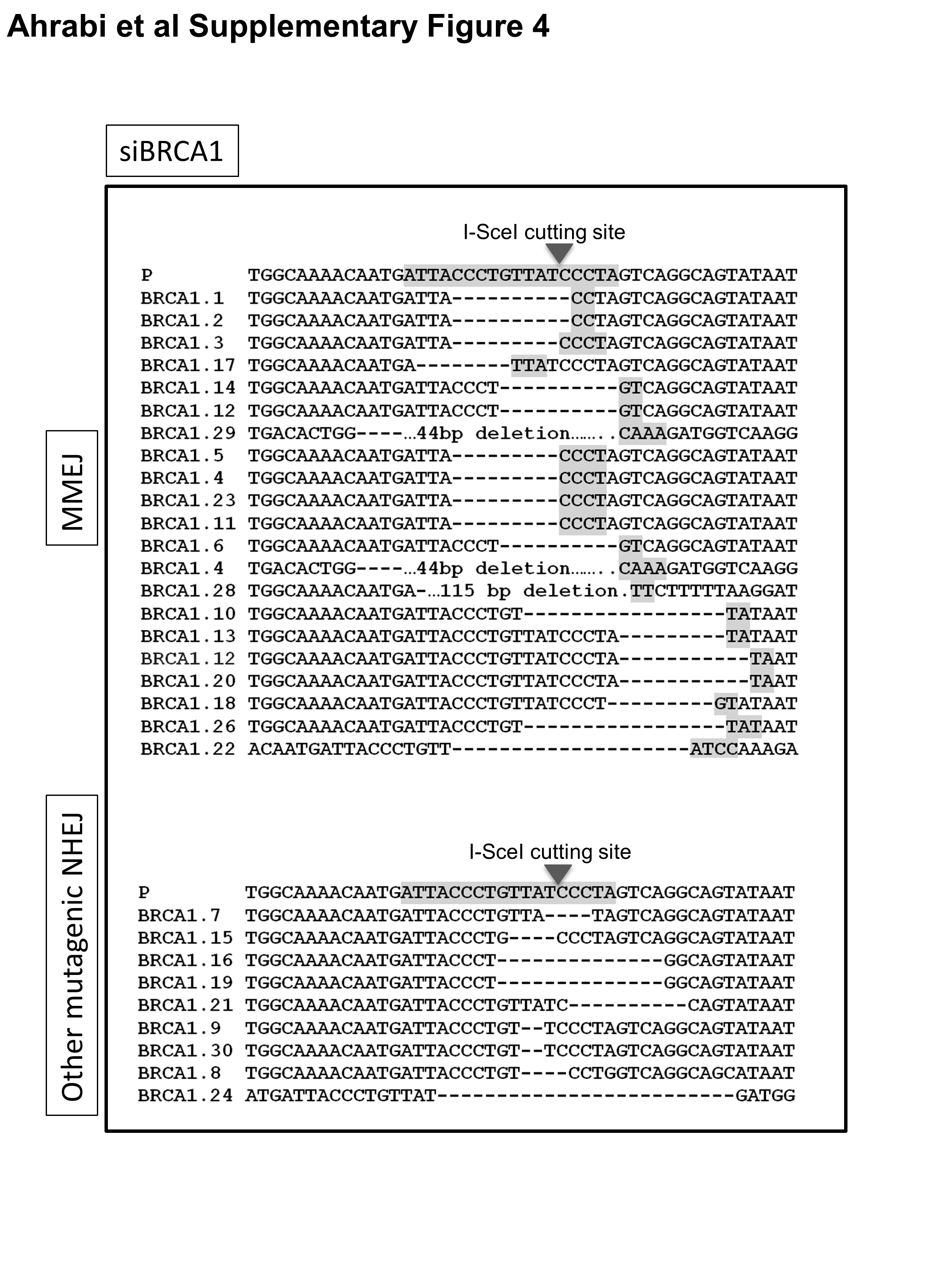

Supplement: SUPPLEMENTARY DATA [file supp_gkw326_nar-03361-d-2015-File009.zip › FigS6.TIF]

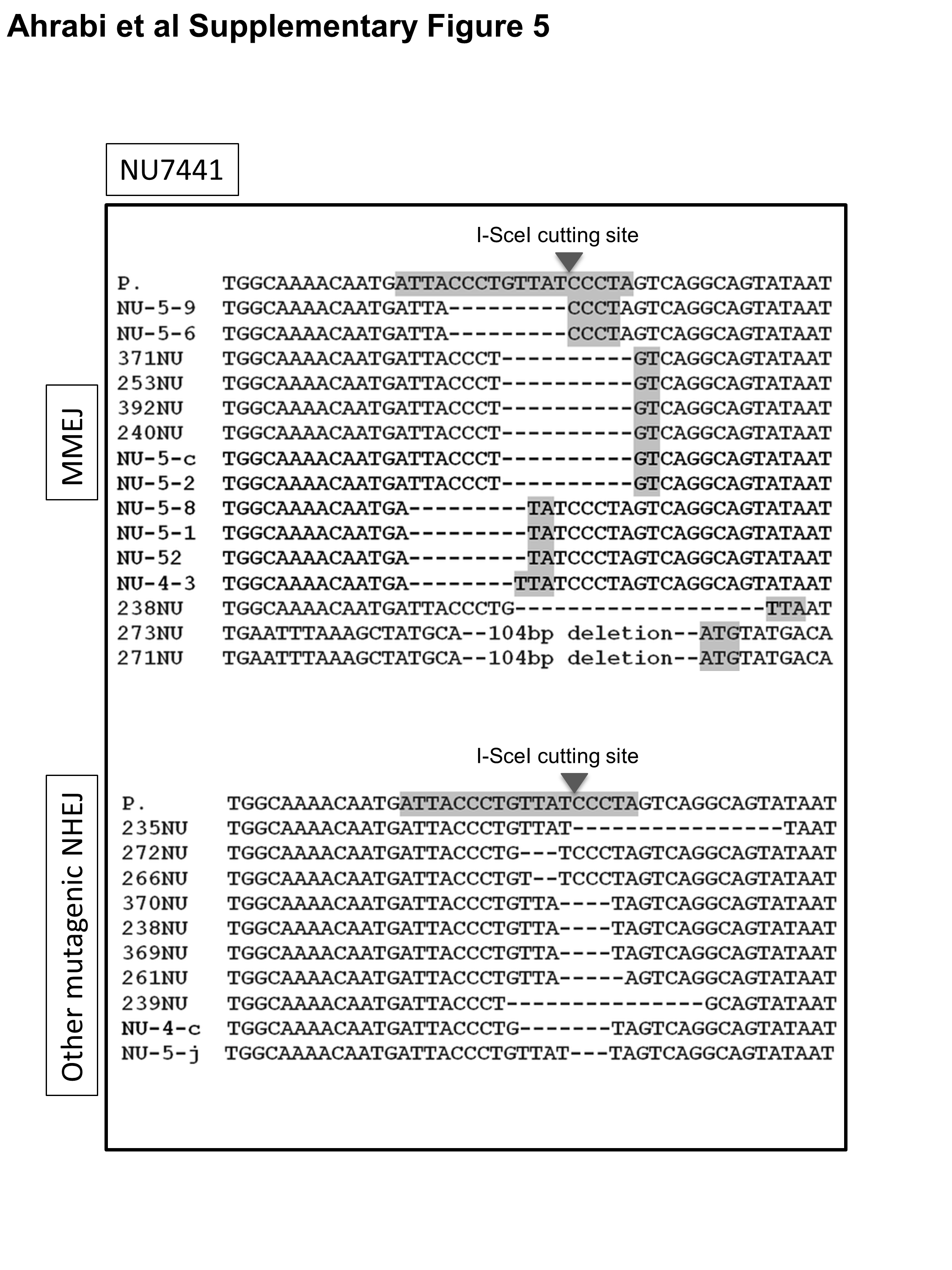

Supplement: SUPPLEMENTARY DATA [file supp_gkw326_nar-03361-d-2015-File009.zip › FigS7.TIF]

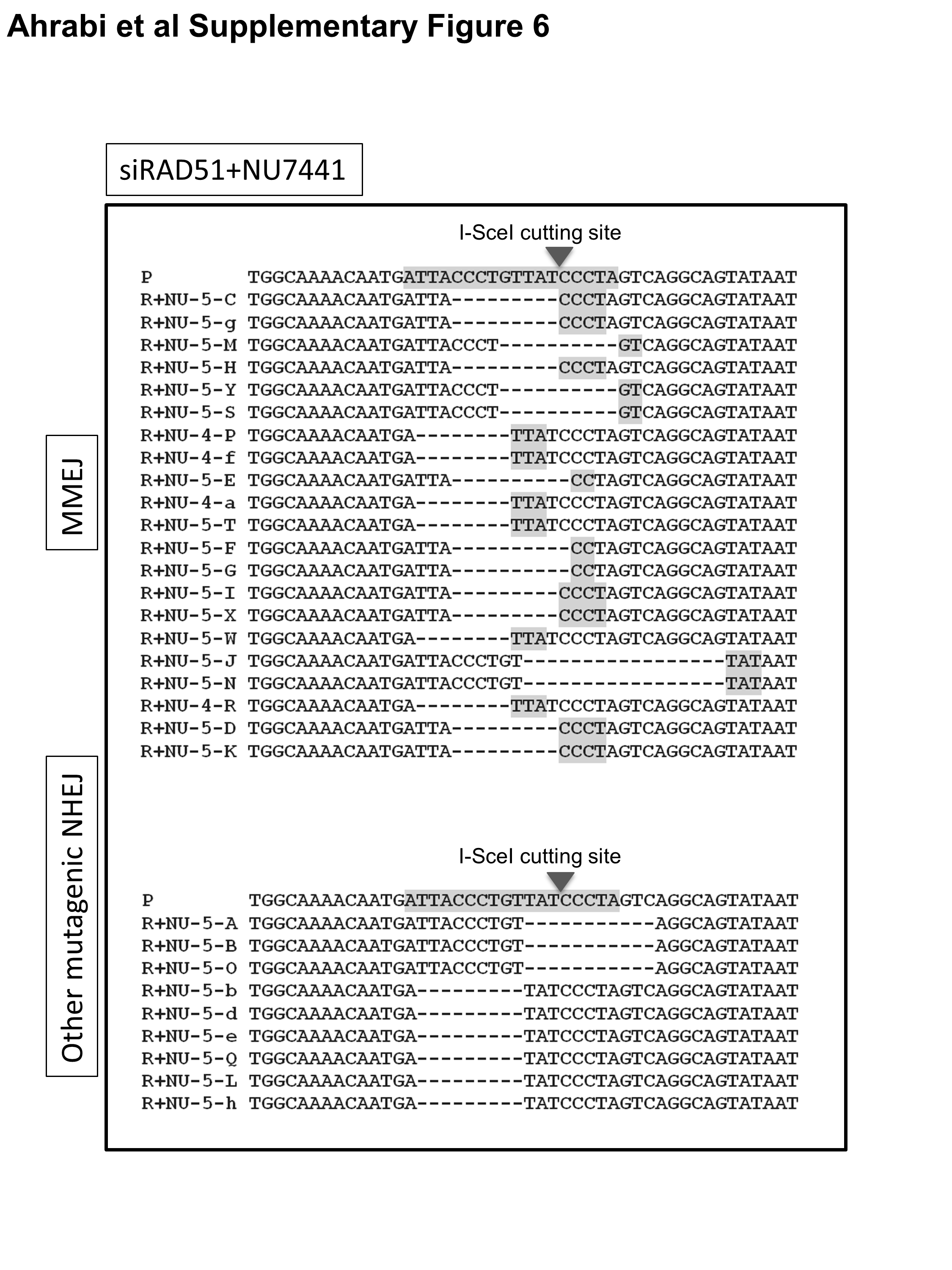

Supplement: SUPPLEMENTARY DATA [file supp_gkw326_nar-03361-d-2015-File009.zip › FigS8.TIF]

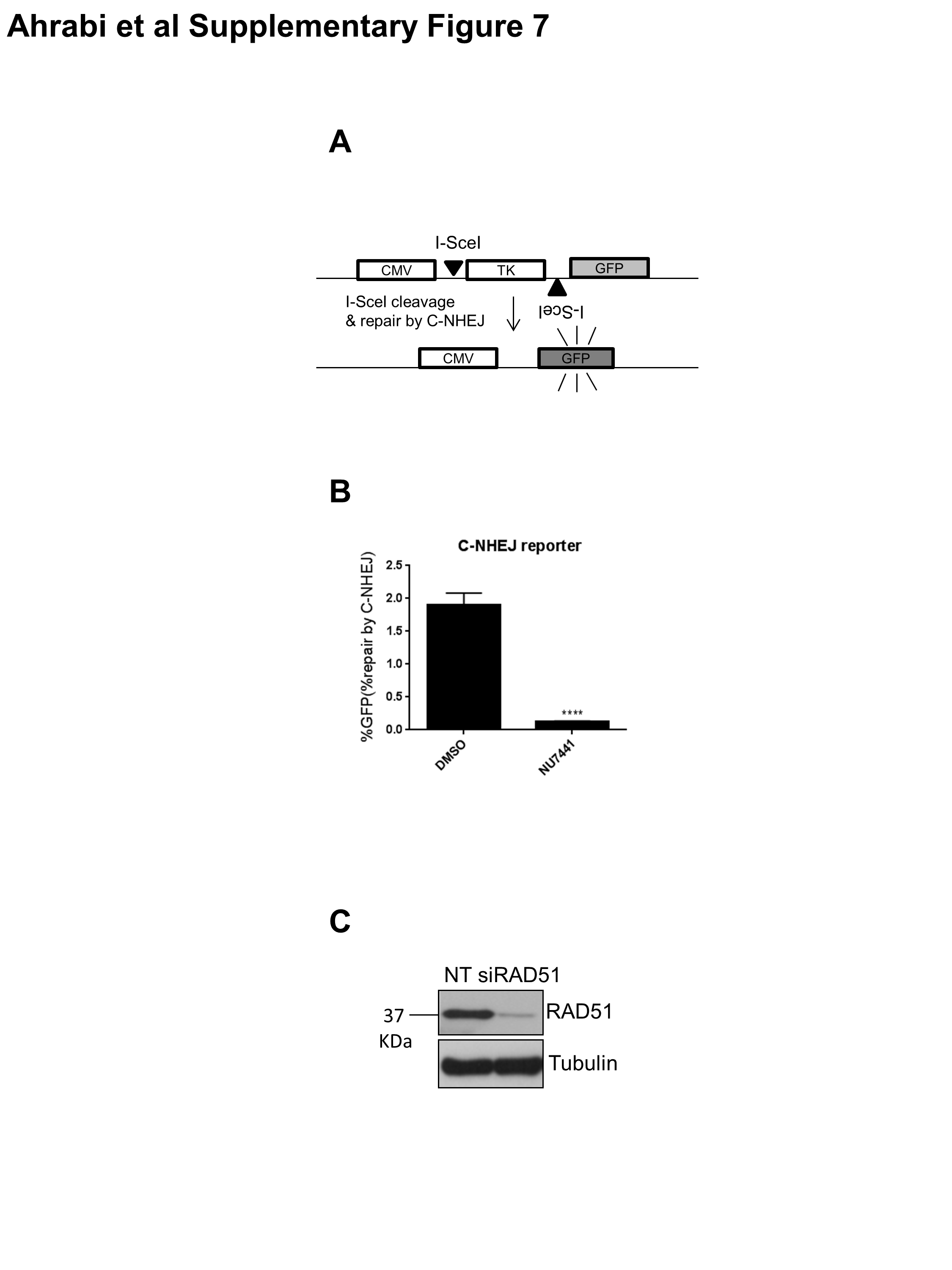

Supplement: SUPPLEMENTARY DATA [file supp_gkw326_nar-03361-d-2015-File009.zip › FigS9.TIF]

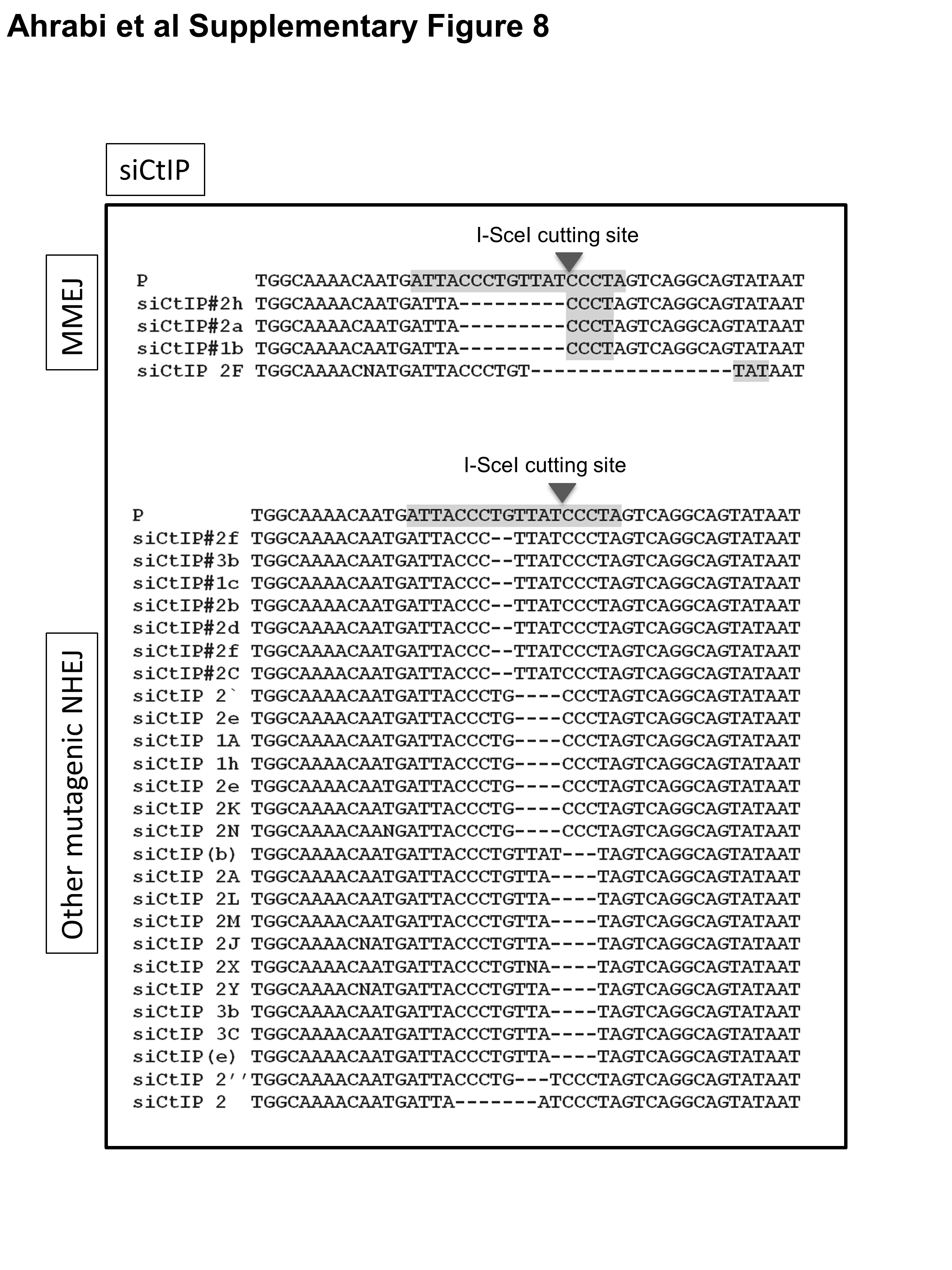

Supplement: SUPPLEMENTARY DATA [file supp_gkw326_nar-03361-d-2015-File009.zip › FigS10.TIF]

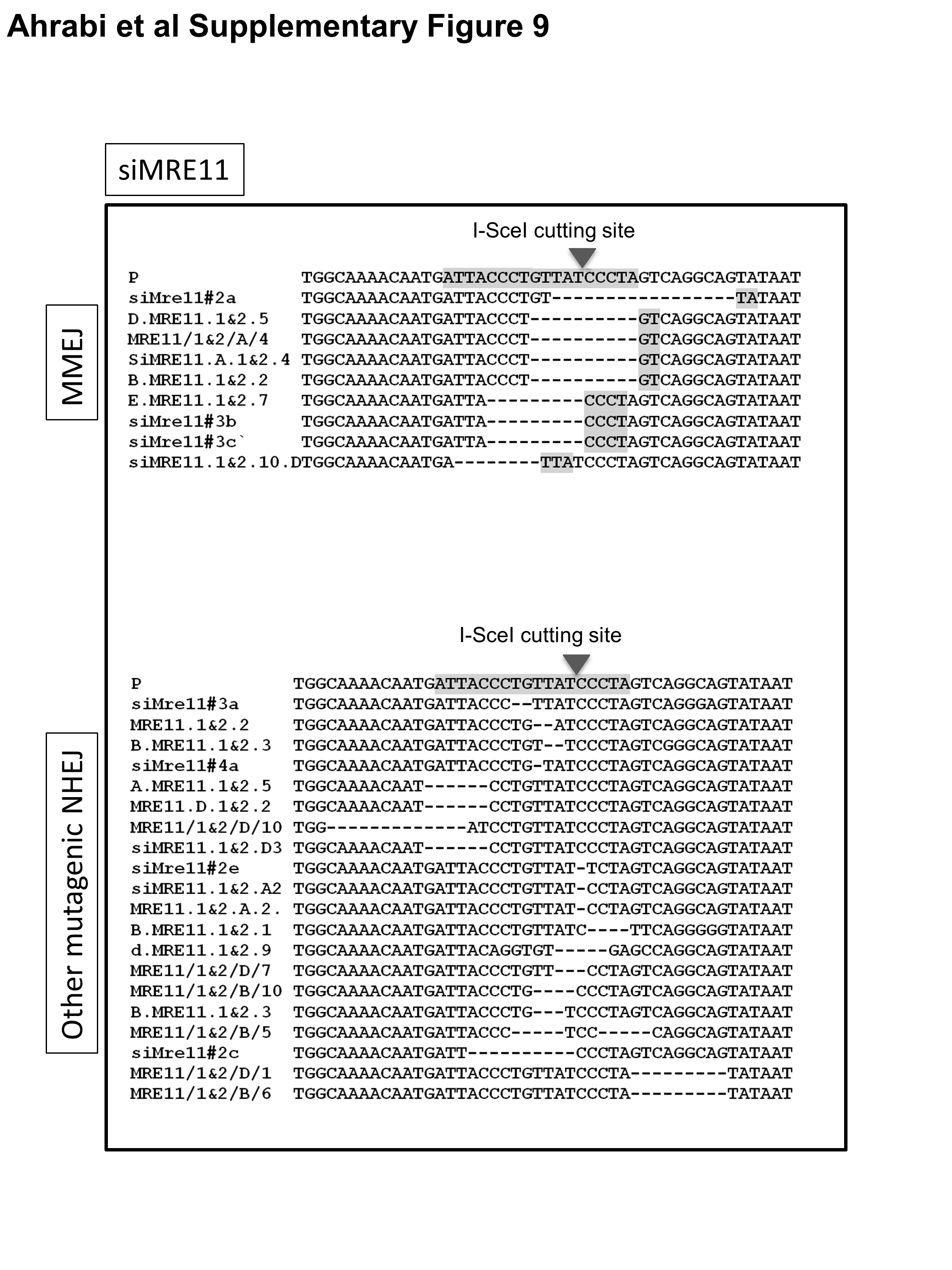

Supplement: SUPPLEMENTARY DATA [file supp_gkw326_nar-03361-d-2015-File009.zip › FigS11.TIF]

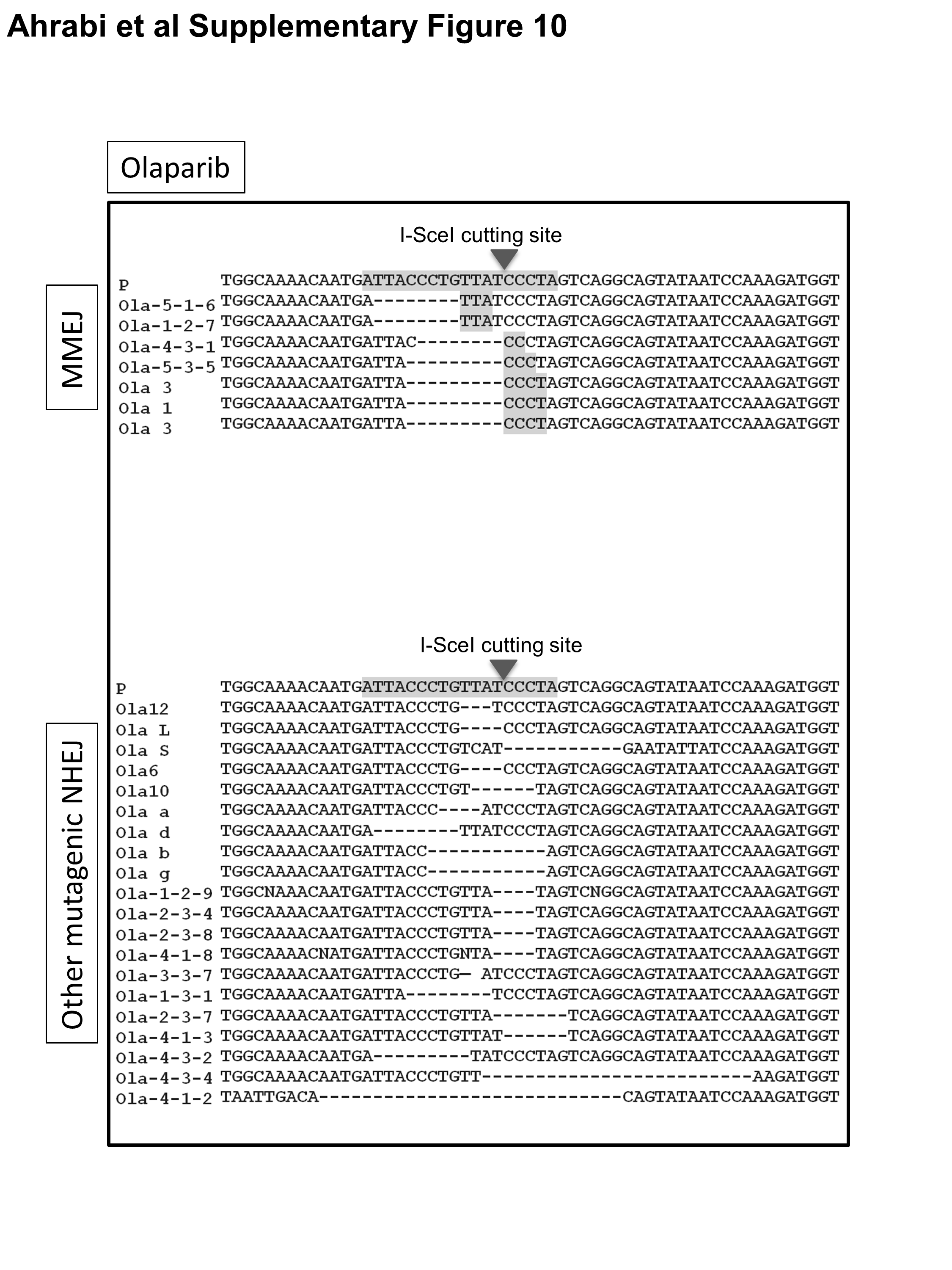

Supplement: SUPPLEMENTARY DATA [file supp_gkw326_nar-03361-d-2015-File009.zip › FigS12.TIF]

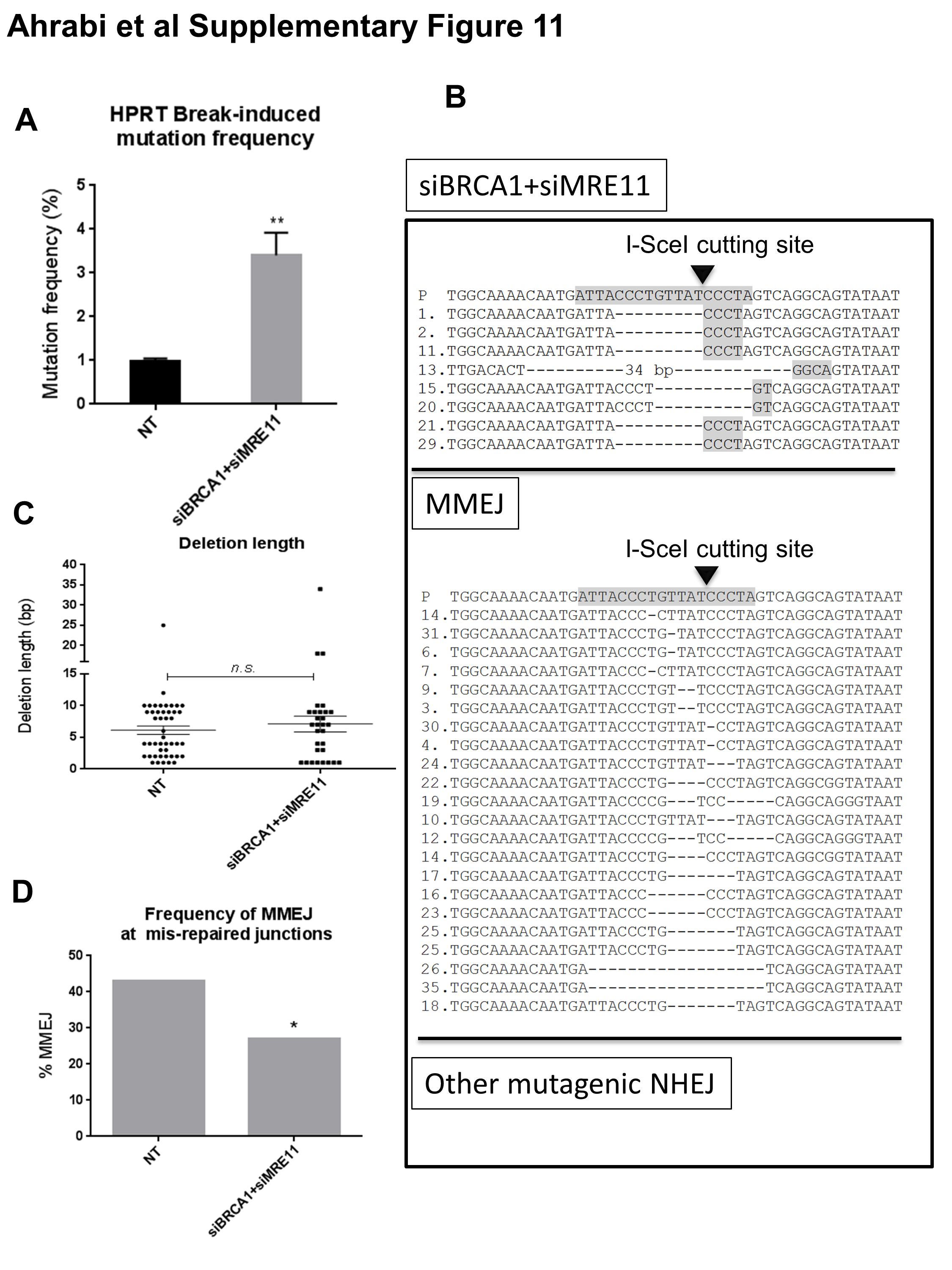

Supplement: SUPPLEMENTARY DATA [file supp_gkw326_nar-03361-d-2015-File009.zip › FigS13.TIF]

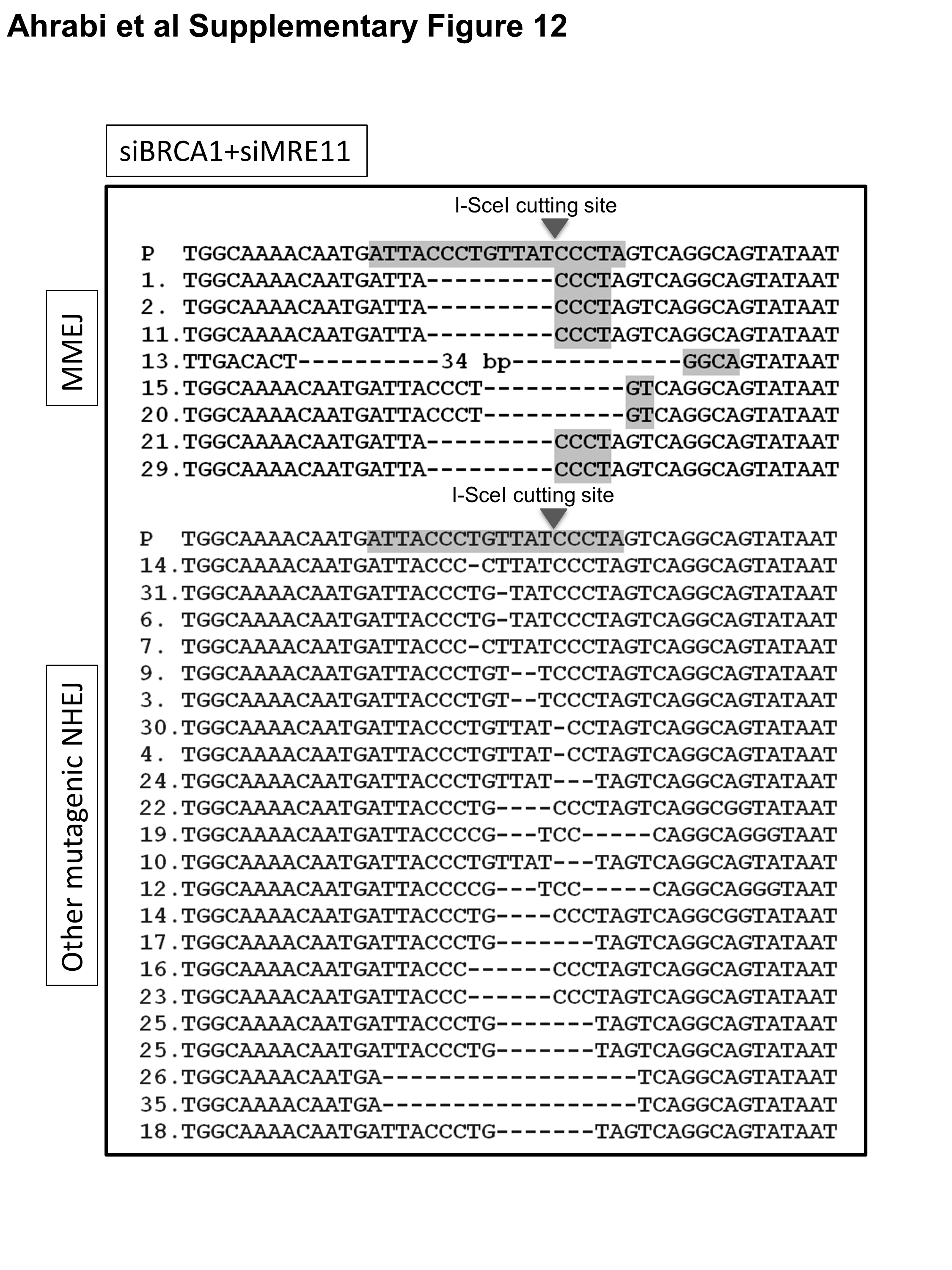

Supplement: SUPPLEMENTARY DATA [file supp_gkw326_nar-03361-d-2015-File009.zip › FigS14.TIF]

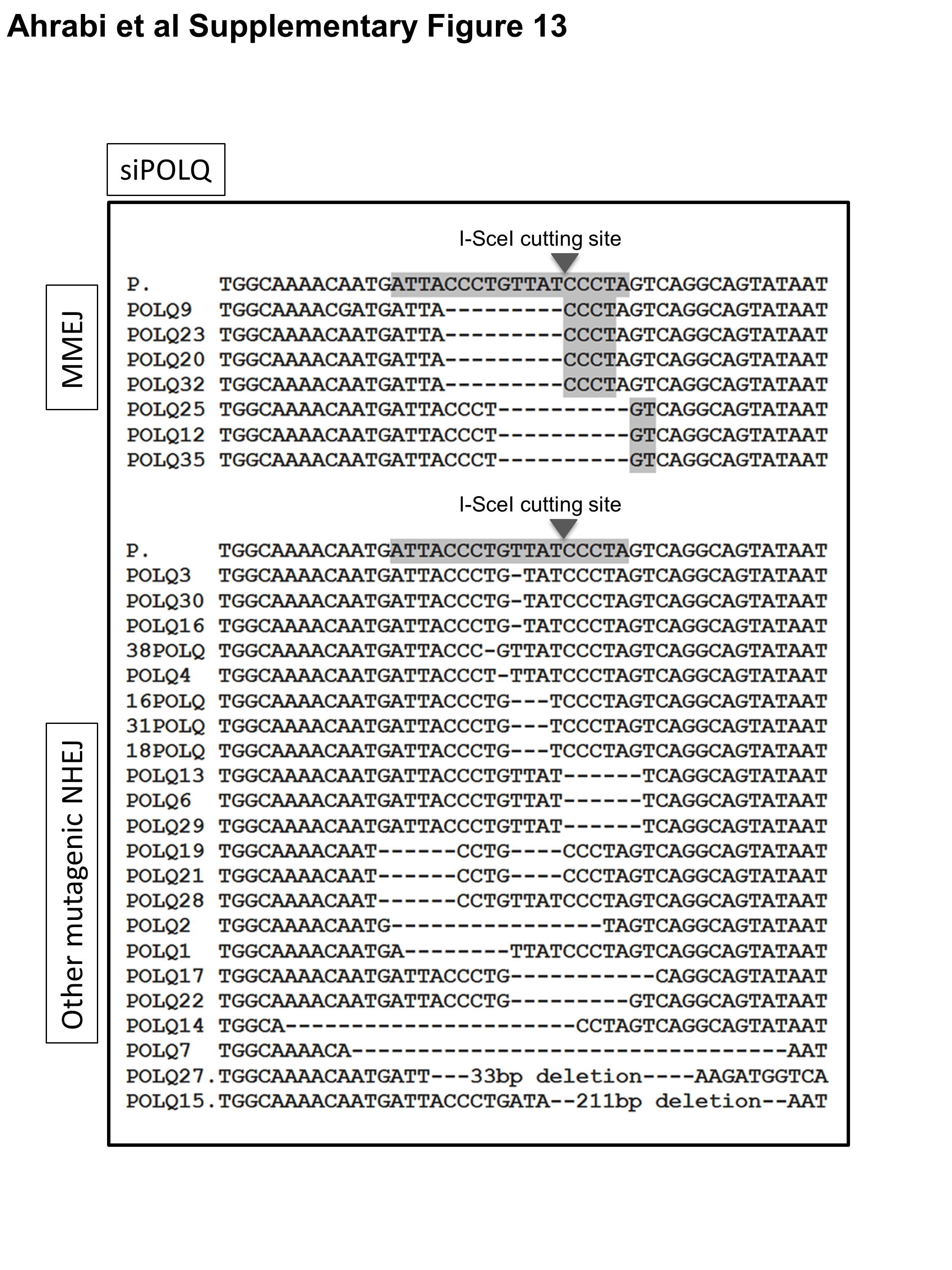

Supplement: SUPPLEMENTARY DATA [file supp_gkw326_nar-03361-d-2015-File009.zip › FigS15.TIF]
